# A real-time framework for mapping subsea cable burial state using Poincaréspectral coherence of DAS measurements

**DOI:** 10.1038/s41598-026-59846-4

**Published:** 2026-08-03

**Authors:** Hamid Shiri, Mohammad Belal

**Affiliations:** 1 National Oceanography Centre, European Way, Southampton, UK; 2 Department of Electronics and Computer Science, Southampton, UK

**Keywords:** Ocean sciences, Physics, Solid Earth sciences

## Abstract

Distributed acoustic sensing (DAS) on subsea fibre-optic cables is emerging as a powerful tool for underwater acoustics, providing dense, kilometre-scale measurements of sound propagation through the water column, the seabed, and the cable’s ambient environment. These observations enable new approaches to environmental acoustic monitoring and subsea-infrastructure assessment, including the detection of oceanographic processes, anthropogenic noise, and geophysical wavefields. However, a central challenge remains: fidelity of DAS measurements depends critically on acoustic coupling between the cable and its surroundings, i.e., variations in burial, exposure, and suspension alter the incident acoustic energy coupling into the fibre, introducing inconsistencies or artefacts in environmental and structural interpretations. Detecting these coupling states directly from DAS data is difficult because the signatures are subtle and datasets are exceptionally large. We introduce a simple, scalable method based on Poincaré spectral coherence. It quantifies the consistency of neighbouring channels across selected acoustic frequency bands. Buried segments show smooth, coherent spectral behaviour, whereas exposed or suspended sections exhibit sharp spatial variability. Applied to two shallow-water deployments, including a 5.8-km coastal cable with diver-verified burial, the method reliably identifies major coupling transitions. Its unsupervised, computationally efficient, real-time compatibility strengthens the case for DAS as a next-generation underwater vibrations sensing technology.

## Introduction

Distributed acoustic sensing (DAS) is rapidly transforming marine geoscience and ocean observing by converting existing subsea fibre-optic cables into dense, kilometre-scale sensor arrays capable of estimating axial strain or strain-rate (via differential optical phase or phase-rate changes) at metre-scale resolution over hundreds of kilometres^1,2^. This enables continuous monitoring of diverse ocean and seafloor processes, including microseisms^3,4^, surface gravity waves^5,6^, internal tides^7,8^, near-bottom turbulence^9^ and anthropogenic activity such as vessel passages and ship noise^10–16^. DAS has also demonstrated sensitivity to marine mammals vocalizations^17,18^, highlighting its potential for ecological monitoring alongside hazard detection and environmental surveillance^19,20^. However, extracting reliable information from DAS data requires robust processing methods capable of operating under highly non-stationary forcing and terabyte-scale data volumes^1^. Classical time–frequency methods remain central in this context^21–26^., including applications to noise chracterisation^27^, turbulence analysis^9^ and vessel tracking^16^, while array-processing techniques^28,29^and modern machine-learning approaches^30–37^are increasingly explored.

A critical limitation in subsea DAS observation is that recorded signals are strongly modulated by local cable–seafloor coupling. Cable segments may be buried, partially buried, exposed, or suspended, and each state responds differently to identical forcing. Burial increases effective mass, stiffness, and damping through sediment confinement, suppressing mid- to high-frequency motion and producing a low-pass–like response, whereas exposed or suspended spans can exhibit enhanced broadband sensitivity or resonant vibration driven by waves, currents, turbulence, trawling, and vessel activity. Consequently, the same physical process may manifest very differently along a single cable, complicating interpretation of wavefields, ambient noise, and anthropogenic signatures. Recent studies confirm the importance of coupling, for example, Bakulin et al.^38^show that sub-wavelength variations in seabed stiffness can produce strong frequency-dependent amplitude modulation in seafloor DAS observations, highlighting that coupling effects may depend on the dominant wave type and mode content (e.g., Scholte, Rayleigh, or body-wave contributions) while, Taweesintananon et al^39^. demonstrate that changes in coupling can shift near-surface shear-wave resonance frequencies, suggesting that resonance behaviour itself can provide an independent proxy for burial state. Despite this, no automated framework currently provides real-time, spatially continuous mapping of burial or coupling state along subsea fibre-optic cables. Harmon et al^10^. showed coupling-dependent frequency-band-energy patterns in vessel signals, but the approach relied on manual interpretation and strong anthropogenic excitation, limiting applicability for continuous monitoring. Other studies reported sensitivity changes linked to substrate variability or cable contact^40,41^ but did not quantify coupling or burial transitions or produce segment-resolved coupling maps.

Here we address this gap by investigating whether cable–seafloor coupling state can be inferred from the spatial regularity of DAS-derived spectral features. Motivated by previous observations that cable coupling influences the spatial and spectral characteristics of DAS measurements, we hypothesise that well-coupled segments (often associated with burial) will tend to exhibit smoother and more coherent behaviour across neighbouring channels, whereas weakly coupled, exposed, or free-span segments may exhibit greater spatial irregularity. This motivates the use of smoothness-based metrics as indicators of coupling-state variability along the cable using a Poincaré-type spectral coherence metric. We compute two-band spectral fingerprints and evaluate their spatial irregularity using block-wise Poincaré descriptors combined with robust normalisation and spatial smoothing. The resulting coherence field enables computationally efficient, near-real-time mapping of coupling transitions along the cable, distinguishing buried, partially buried, exposed, and suspended segments without requiring controlled sources, vessel passes, or prior geological knowledge.

Summary of novel contributions comprise:

- A physically interpretable and automated framework for real-time mapping of subsea cable burial/coupling state from DAS recordings, producing spatially continuous segment-resolved profiles.
- A lightweight Poincaré spectral coherence metric that quantifies coupling irregularity using two-band spectral fingerprints and remains robust under low-amplitude, strongly time-varying forcing.
- A scalable near-real-time workflow based on robust normalisation and spatial smoothing, enabling operational deployment without reliance on computationally intensive machine-learning models or high-performance computing infrastructure.

**Paper structure**

The remainder of this paper comprises: Section Methodology which presents the proposed real-time burial-state mapping framework, including spectral fingerprint construction, the Poincaré spectral-coherence metric, and robust normalization and smoothing. Section Real data analysis which describes the European Marine Energy Centre (EMEC) Eday and Observatory of the Sea (OBSEA) datasets and presents the resulting burial or ambient coupling state maps. Section Discussion which discusses implications, limitations, and future refinements. Section Conclusion which concludes and outlines future work, including integration into real-time processing pipelines and extension to longer and more diverse subsea cables.

**Fig. 1:**
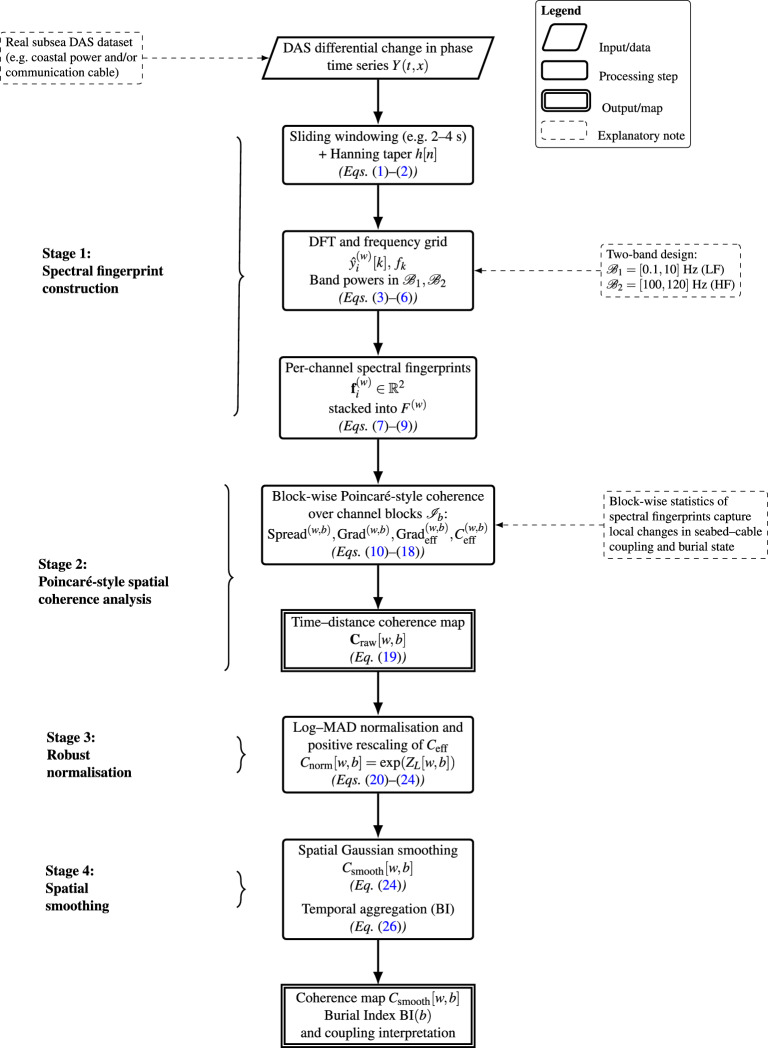
Workflow of the spectral fingerprint and Poincaré-style coherence framework for DAS data. **Stage 1:** windowing, tapering, and frequency-domain transformation to form two-band spectral fingerprints. **Stage 2:** block-wise Poincaré statistics yielding the coherence index $C_{\textrm{eff}}^{(w,b)}$ and a time–distance map. **Stage 3:** log–median–absolute–deviation (MAD) normalisation and rescaling. **Stage 4:** Spatial smoothing to derive $C_{\textrm{smooth}}$ and BI for burial-state identification.

## Methodology

This section presents the complete processing pipeline used to transform DAS measurements (derived from differential optical phase-change) into a spatially resolved indicator of seabed–cable coupling for detecting buried, exposed, and suspended segments of subsea cables. The framework is designed to operate in real time and is organised into four sequential stages, each addressing a key physical or computational challenge associated with DAS-based cable monitoring.

In the first stage, the raw DAS signals are converted into compact spectral fingerprints that summarise the low- and high-frequency energy in short time windows. This enables the separation of gravity-wave-driven low-frequency motion from the relatively higher-frequency responses associated with turbulent flow, mechanical vibration, or vessel activity–two regimes that behave distinctly depending on whether the cable is buried or unburied.

In the second stage, Poincaré-style spatial coherence statistics are computed along neighbouring channels. Buried cable typically exhibits smooth, coherent spectral behaviour across space, whereas unburied or suspended segments show abrupt channel-to-channel fluctuations. By quantifying both the within-block variability and the local gradient of spectral fingerprints, this stage captures the essential physical contrast between these cable–seabed coupling states.

In the third stage, the resulting coherence values are normalised using median–absolute–deviation (MAD) to remove large-scale temporal variations introduced by tides, environmental forcing, or interrogator drift. This ensures that the coherence index reflects true spatial transitions along the cable rather than global changes in signal amplitude.

Finally, a spatial Gaussian smoothing operation is applied to the normalised coherence field to produce a geophysically interpretable time–distance coherence map. This smoothing suppresses channel-scale artefacts, enhances spatial continuity, and emphasises burial-scale structure, thereby highlighting transitions between buried and unburied regions.

The resulting smoothed Poincaré spectral coherence map is subsequently aggregated in time to derive the BI (burial index), which provides a stable one-dimensional indicator of spatial coupling variability along the cable. Together, these representations offer a robust and interpretable framework for real-time cable monitoring and geophysical interpretation.

The overall workflow of the proposed method is illustrated in Fig. 1, and each processing stage is discussed in detail in the following subsections.

### Spectral fingerprint construction

The DAS differential phase-change measurements, *Y*(*t*, *x*), are partitioned into overlapping analysis windows of length $N_t^{(w)}$, where *w* denotes the window index. Within each window, the time and channel indices are represented by *n* and *i*, respectively. Spectral fingerprints are computed using short overlapping windows of 2–4 s duration (depending on dataset sampling rate and the required frequency resolution), enabling tracking of rapidly varying high-frequency excitation while maintaining sufficient spectral stability for robust band-power estimation.

Within each window, a Hanning taper is applied to mitigate spectral leakage introduced by sharp temporal truncation. The tapered signal is then transformed to the frequency domain using the discrete Fourier transform (DFT), yielding the complex spectrum $\hat{y}_i^{(w)}[k]$ used for subsequent band-limited energy estimation. Eqs. (1)–(4) formally define these preprocessing and spectral estimation steps.

1 $$\begin{aligned} h[n] = \frac{1}{2}\left( 1 - \cos \left( \frac{2\pi n}{N_t^{(w)} - 1}\right) \right) , \qquad 0 \le n < N_t^{(w)}, \end{aligned}$$

2 $$\begin{aligned} \tilde{y}_i^{(w)}[n] = y_i^{(w)}[n] \, h[n], \end{aligned}$$

3 $$\begin{aligned} \hat{y}_i^{(w)}[k] = \sum _{n=0}^{N_t^{(w)} - 1} \tilde{y}_i^{(w)}[n]\, e^{-j2\pi nk/N_t^{(w)}}, \end{aligned}$$

4 $$\begin{aligned} f_k = \frac{k}{N_t^{(w)}}\, f_s, \end{aligned}$$

Where *k* denotes the discrete frequency-bin index of the DFT (not spatial wavenumber).

Because cable response exhibits frequency-dependent characteristics, we extract energy in two frequency bands. To construct the two-band spectral fingerprints used in the Poincaré-style coherence analysis, frequency bands were selected empirically through systematic inspection of DAS spectra across both datasets, with the aim of maximising spatial contrast and temporal stability in the coherence metric while maintaining robustness to transient events and instrumental noise.

The use of low- and high-frequency bands is further motivated by the fact that coupling state and sediment confinement strongly modulate the frequency response of seafloor DAS signals. Recent studies demonstrate that variations in seabed stiffness and coupling conditions can introduce pronounced frequency-dependent amplitude modulation and resonance shifts, implying that burial state may be inferred from band-specific spectral behaviour^38,39^.

The low-frequency band (0.01–10 Hz) captures persistent, large-scale environmental forcing, including surface gravity waves, swell, and tidal modulation, which induce spatially smooth and temporally stable responses along the fibre and therefore provide a consistent background reference. In contrast, the high-frequency band (100–120 Hz) captures intermittent excitation, including anthropogenic sources (e.g., vessel harmonics) and cable-related mechanical vibration (e.g., current-driven strumming or vortex-induced vibration). The high-frequency components are strongly attenuated by sediment damping when the cable is buried, making this band particularly sensitive to coupling state.

Importantly, the high-frequency band is not assumed to contain continuously coherent wave energy; rather, it reflects episodic narrowband or broadband activity that becomes increasingly suppressed under strong cable–seabed coupling. In addition, this band may see influence, in certain DAS systems, from residual phase-fading effects (Rayleigh fading), i.e., localised signal dropouts caused by destructive interference of the coherent Rayleigh backscatter, which reduce sensitivity and locally degrade the signal-to-noise ratio in affected fibre sections. Despite this, the relative contribution of the high-frequency content provides a robust discriminator of spatial variability in coupling conditions.

Although the exact optimal frequency ranges may vary between deployments due to differences in environmental forcing, sediment properties, cable construction, sensing geometry, and interrogator response, the underlying two-band coherence framework is general. The two-band fingerprint approach therefore provides a flexible and transferable mechanism for capturing coupling-sensitive spectral contrast, and can be readily adapted to alternative frequency intervals without modification of the core methodology. To quantify the relative contribution of the two selected frequency bands, we define a normalized spectral representation based on their respective band powers.

5 $$\begin{aligned} B_1 = [0.1, 10]~\textrm{Hz}, \qquad B_2 = [100, 120]~\textrm{Hz}. \end{aligned}$$

Let $K_k$ be the set of frequency-bin indices that fall inside band $B_k$. The band-limited energy for channel *i* in window *w* is then computed as

6 $$\begin{aligned} P_{i,k}^{(w)} = \sum _{\ell \in K_k} \left| \hat{y}_i^{(w)}[\ell ] \right| ^2, \end{aligned}$$

which corresponds to the spectral power contained within frequency band $B_k$.

To quantify the band-limited spectral content of the DAS response, we define the Frequency Band Energy (FBE) for each channel and time window as the band-integrated spectral power given by Eq. (6). This corresponds to summing the discrete power spectrum, or equivalently the periodogram estimate of the power spectral density, over the selected frequency interval, as detailed in Appendix A. In the discrete short-time Fourier setting used here, FBE is proportional to the variance (power) of the signal contribution within the selected band, and is therefore interpreted as a band-power attribute rather than absolute physical energy.

This band-integrated metric provides a compact measure of how the variance (power) of the DAS response is distributed across physically meaningful frequency bands and is widely used for characterising random processes and broadband noise fields^42^.

Related FBE-style approaches based on time-domain bandpass filtering followed by envelope extraction, aimed at capturing amplitude modulation within specific frequency bands, have previously been applied to DAS observations of shallow-water subsea cables^10^.

To construct a compact representation of the spectral behaviour of each channel, we normalise the two band energies. The total energy across the bands is

7 $$\begin{aligned} S_i^{(w)} = P_{i,1}^{(w)} + P_{i,2}^{(w)} + \varepsilon _1, \end{aligned}$$

where the small constant $\varepsilon _1$ prevents division by zero.

The stabilisation constant $\varepsilon _1$ is introduced solely to avoid division by zero in rare cases where both frequency bands contain negligible energy within a window (e.g., due to local fading or exceptionally quiet conditions). When signal power is non-zero, $\varepsilon _1$ has negligible influence on the resulting normalised fingerprint. Also, As we mentioned throughout this work, small stabilization constants are introduced to prevent division by zero or undefined logarithms. For clarity, we denote these as $\epsilon _1$,$\epsilon _2$, etc., noting that their numerical values may differ depending on context but do not affect relative spatial interpretation.

The spectral fingerprint is then defined as

8 $$\begin{aligned} f_i^{(w)} = \frac{1}{S_i^{(w)}} \begin{bmatrix} P_{i,1}^{(w)} \\ P_{i,2}^{(w)} \end{bmatrix}, \end{aligned}$$

a two-dimensional feature vector encoding the relative contributions of low- and high-frequency energy. Collecting the fingerprints from all $N_{ch}$ channels yields the matrix

9 $$\begin{aligned} F^{(w)} \in \mathbb {R}^{N_{ch} \times 2}, \end{aligned}$$

which serves as the input for the subsequent spatial coherence analysis.

### Poincaré-style spatial coherence

Cable coupling to the seabed typically varies smoothly over buried or well-coupled to seafloor) regions but exhibits abrupt transitions at exposed or suspended segments. To capture this behaviour, the cable is divided into spatial blocks of *M* consecutive channels,

10 $$\begin{aligned} \mathscr {I}_b = \{i_b,\, i_b+1,\, \ldots ,\, i_b + M - 1\}, \end{aligned}$$

where *b* indexes the block along the cable. Note that: in this work, a DAS channel refers to a single spatial sampling point along the fibre (with spacing *Δ x*). A channel block refers to a contiguous set of M neighbouring channels used to compute local spatial statistics (spread, gradient, and coherence) over a windowed interval. Blocks are indexed by b and may overlap depending on the chosen step size. Within block $\mathscr {I}_b$, the average spectral fingerprint is computed as

11 $$\begin{aligned} \bar{f}^{(w,b)} = \frac{1}{M} \sum _{i \in \mathscr {I}_b} f_i^{(w)}. \end{aligned}$$

This average represents the typical spectral response of channels within the block.

The spatial behaviour captured by the proposed framework is rooted in the physics of cable–seabed coupling. Previous studies have demonstrated that DAS measurements are strongly modulated by the degree of mechanical coupling between the cable and its environment. In exposed configurations, the response is dominated by hydrodynamic pressure forcing, while buried or partially buried cables are subject to additional shear and normal stresses within the sediment, resulting in more structured and spatially coherent signals. Conversely, weakly coupled or suspended segments exhibit increased variability and irregular spectral behaviour^10^.

The degree to which individual channel fingerprints deviate from this mean is quantified by the spectral spread, defined as an $L_2$ norm over the channel space,

12 $$\begin{aligned} \textrm{Spread}_{L_2}^{(w,b)} = \sqrt{ \frac{1}{M} \sum _{i \in \mathscr {I}_b} \left\| f_i^{(w)} - \bar{f}^{(w,b)} \right\| _2^2 }. \end{aligned}$$

This quantity increases when spectral fingerprints fluctuate irregularly or abruptly across the block. Such behaviour is expected in unburied, poorly coupled or partially suspended segments because the cable is less mechanically constrained by the seabed and may exhibit localised free-span dynamics, including bending, intermittent sediment contact, and vortex-induced vibration driven by near-bottom currents and turbulent flow. Exposed segments are also more directly excited by water-column disturbances and vessel-generated forcing, leading to spatially heterogeneous boundary conditions and stronger channel-to-channel variability. These coupling-dependent effects can produce abrupt changes in the observed response across adjacent channels^10,38–40^.

In addition, channel-to-channel variability is characterised through the discrete spectral gradient

13 $$\begin{aligned} \Delta f_i^{(w)} = f_{i+1}^{(w)} - f_i^{(w)}, \end{aligned}$$

from which the block-level gradient magnitude is defined as an $L_2$ norm across neighbouring channels,

14 $$\begin{aligned} \textrm{Grad}_{L_2}^{(w,b)} = \sqrt{ \frac{1}{M-1} \sum _{i \in \mathscr {I}_b\setminus \{\textrm{last}\}} \left\| \Delta f_i^{(w)} \right\| _2^2 }. \end{aligned}$$

The gradient captures how rapidly the spectral fingerprints vary from one channel to the next.

Conversely, buried (or well coupled) segments are embedded within sediment (or well supported) and are therefore subject to increased mechanical confinement and damping. This reduces the degrees of freedom of cable motion and suppresses rapid channel-to-channel variability, such that neighboring channels experience similar effective boundary conditions and exhibit smoother, more spatially coherent spectral fingerprints.

To avoid artificially large coherence values in regions where both spread and gradient are extremely small, a gradient floor proportional to the typical energy level in the block is introduced.

15 $$\begin{aligned} \textrm{Scale}_{L_2}^{(w,b)} = \sqrt{ \frac{1}{M} \sum _{i \in \mathscr {I}_b} \Vert f_i^{(w)} \Vert _2^2 }, \end{aligned}$$

and the gradient floor is given by

16 $$\begin{aligned} \textrm{Grad}_{\textrm{floor}}^{(w,b)} = \alpha \, \textrm{Scale}_{L_2}^{(w,b)}, \qquad \alpha = 0.05, \end{aligned}$$

leading to the effective gradient

17 $$\begin{aligned} \textrm{Grad}_{\textrm{eff}}^{(w,b)} = \max \!\left( \textrm{Grad}_{L_2}^{(w,b)},\, \textrm{Grad}_{\textrm{floor}}^{(w,b)} \right) , \end{aligned}$$

where the parameter *α* controls the minimum effective gradient floor and was selected empirically as *α* = 0.05 to prevent artificial inflation of the coherence ratio in near-uniform blocks. Sensitivity testing showed that the resulting burial index is stable for *α* values in the range 0.01–0.1, indicating that the method is not strongly dependent on fine tuning of this parameter.

The effective Poincaré-style spatial coherence index is then defined as

18 $$\begin{aligned} C_{\textrm{eff}}^{(w,b)} = \frac{ \textrm{Spread}_{L_2}^{(w,b)} }{ \textrm{Grad}_{\textrm{eff}}^{(w,b)} + \varepsilon _2 }, \end{aligned}$$

where $\varepsilon _2$ is small stabilization constant that are introduced to prevent division by zero or undefined logarithms.

The structure of $C_{\textrm{eff}}$ is motivated by Poincaré-type inequalities, which relate the variability of a function over a domain to the magnitude of its spatial gradient. Here, the fingerprint spread quantifies within-block variability, while the gradient term measures the characteristic channel-to-channel change. Their ratio therefore provides a dimensionless measure of spatial irregularity. Larger values of $C_{\textrm{eff}}$ indicate that neighbouring channels exhibit comparatively dissimilar spectral fingerprints, whereas smaller values correspond to smoother and more spatially consistent behaviour. Because cable–seafloor coupling influences the spatial regularity of the measured DAS response, $C_{\textrm{eff}}$ provides a practical indicator of coupling state and its potential relationship to burial conditions. This interpretation is treated as a physically motivated hypothesis and is subsequently evaluated using field observations and independent geological context.

Aggregating the coherence estimates across all time windows yields the raw time–distance coherence map,

19 $$\begin{aligned} \textbf{C}_{\textrm{raw}}[w,b] = C_{\textrm{eff}}^{(w,b)}, \end{aligned}$$

which provides a first-order, unsupervised representation of spatial variability along the cable. We emphasise that the proposed coherence metric is fundamentally sensitive to cable–seafloor coupling state. Burial is a dominant contributor to coupling variability, but other factors such as sediment heterogeneity, bathymetric gradients and seabed rugosity, protective structures, local free spans, and installation transitions may also influence the observed spatial irregularity^10,38,39^.

### Robust normalisation and spatial smoothing

While the raw Poincaré coefficient $C_{\textrm{eff}}(w,b)$ is strictly non–negative by definition (being a ratio of $L_2$ norms), its amplitude may vary substantially in time due to environmental changes, bursty noise, or large-scale variations in signal energy. To stabilise the dynamic range while preserving positivity and interpretability, we apply a robust log–based normalisation.

First, the raw coefficients are transformed using a logarithm,

20 $$\begin{aligned} L[w,b] = \log \!\big (C_{\textrm{raw}}[w,b] + \varepsilon _3\big ), \end{aligned}$$

which compresses large values and reduces the strong positive skewness of the raw coefficient distribution, which typically contains a small number of extreme values arising from highly exposed/free-span segments or abrupt coupling transitions. The log transform compresses these heavy-tailed outliers while preserving the ordering of the underlying physical coefficient. Also, The small constant $\epsilon _3$ is included solely for numerical stability, preventing division by zero in rare cases where both the within-block spread and the effective gradient become extremely small.

A robust normalisation is then applied to the log–transformed values using the median and MAD,

21 $$\begin{aligned} m_L = \textrm{median}(L), \qquad \textrm{MAD}_L = \textrm{median}\!\big (|L - m_L|\big ), \end{aligned}$$

giving the centred and scaled normalised field

22 $$\begin{aligned} Z_L[w,b] = \frac{ L[w,b] - m_L }{ 1.4826\,\textrm{MAD}_L + \varepsilon _3 }. \end{aligned}$$

The factor 1.4826 is a consistency constant relating the MAD to the standard deviation under Gaussian assumptions (see Appendix B). In this work, it is used as a reference scaling to express the MAD on a comparable scale to the standard deviation. No assumption of normality is imposed on the DAS data; the MAD is adopted due to its robustness to outliers and non-Gaussian behaviour inherent in the measurements.

Finally, to recover a strictly positive and energy–like index while retaining the robust scale normalisation, the values are mapped back to the physical domain via

23 $$\begin{aligned} C_{\textrm{norm}}[w,b] = \exp \!\big (Z_L[w,b]\big ). \end{aligned}$$

Stage 4 of the framework applies spatial smoothing to the normalised coherence field to suppress channel-scale artefacts and enhance geophysically interpretable burial-scale structure. To obtain a geophysically interpretable map, we apply spatial Gaussian smoothing,

24 $$\begin{aligned} C_{\textrm{smooth}}[w,b] = \sum _{k} g_\sigma (b-k)\, C_{\textrm{norm}}[w,k], \end{aligned}$$

where $g_\sigma (\cdot )$ denotes a discrete Gaussian kernel defined as

25 $$\begin{aligned} g_\sigma (\ell ) = \frac{1}{Z} \exp \left( -\frac{\ell ^2}{2\sigma ^2}\right) , \end{aligned}$$

with *ℓ = b - k* and $Z = \sum _{\ell } \exp \left( -\frac{\ell ^2}{2\sigma ^2}\right)$ a normalisation constant ensuring $\sum _{\ell } g_\sigma (\ell ) = 1$. The parameter $\sigma = L_{\textrm{smooth}}/\Delta x$ determines the effective smoothing scale in units of spatial samples, where *Δ x* is the channel spacing. Reflective boundary conditions are applied at the cable ends.

For applications requiring a spatially resolved geological indicator, the two-dimensional coherence field is temporally aggregated to obtain a one-dimensional Burial Index (BI) profile along the cable. Specifically, we define

26 $$\begin{aligned} \textrm{BI}(b) = \textrm{median}_{w} \left( C_{\textrm{smooth}}[w,b] \right) , \end{aligned}$$

where the median is taken over time windows to suppress transient fluctuations and emphasise persistent spatial structure. Also, we tested multiple window lengths spanning short (2–4 s) windows for spectral fingerprint construction and longer aggregation windows to establish stability when ascertaining $C_{\textrm{smooth}}$, in Eq. (24). The spatial structure of BI (representing burial transition locations and persistent regions of elevated and reduced coherence) remains consistent across tested window lengths, while longer windows reduce variance and enhance stability at the expense of temporal responsiveness. This indicates that the method is not strongly sensitive to moderate changes in window duration

The resulting smoothed indicator $C_{\textrm{smooth}}$ preserves the physical interpretation of the Poincaré coherence coefficient (strict positivity and monotonicity with spatial irregularity), while providing robustness to outliers and temporal variability. In essence we use a frequency-resolved Poincaré-type ratio of spatial variance to spatial roughness of spectral fingerprints, with a log–MAD–exp normalization that preserves positivity and physical meaning while stabilizing temporal variability. Consequently, large values correspond to blocks with irregular, weakly constrained motion (typical of exposed or suspended cable sections), whereas small values indicate spatially coherent behaviour characteristic of buried or well-supported segments.

It is important to emphasise that BI does not measure burial state directly, but quantifies spatial irregularity in the cable–seafloor coupling response. Accordingly, the BI should be interpreted fundamentally as a coupling-state indicator, that can be used to ascertain influence from burial, whilst also capturing additional controls such as bathymetric variability, seabed rugosity, sediment heterogeneity, and local free-span conditions.

### Computational considerations

The computational cost of the proposed Poincaré spectral coherence workflow is dominated by the short-time Fourier transform (STFT) used to construct the spectral fingerprints. As summarised in Table 1, this stage scales as $\mathscr {O}(N_w N_{\textrm{ch}} N_t \log N_t)$, where $N_{\textrm{ch}}$ is the number of DAS channels, $N_t$ is the number of samples per time window, and $N_w$ is the number of time windows. Other variable, such as *L* and $N_{\textrm{blk}}$ represents the cable length (or equivalently the total spatial aperture/extent being analysed), typically in metres or kilometres and the number of spatial blocks into which the cable is divided for the block wise Poincaré spectral coherence calculation.

Subsequent stages involve only block-wise averaging, finite-difference gradient computation, robust median/MAD normalisation, and one-dimensional Gaussian smoothing, each of which scales linearly with the number of spatial blocks.

The method therefore requires no iterative optimisation or training and is well suited for continuous real-time implementation.

**Table 1:** Computational complexity of the proposed workflow.

Stage	Main computation	Scaling
Stage 1: Spectral fingerprint	FFT + band power	$\mathscr {O}(N_w\,N_{\textrm{ch}}\,N_t \log N_t)$
Stage 2: Poincaré coherence	Spread + gradient	$\mathscr {O}(N_w\,N_{\textrm{blk}}\,M)$
Stage 3: Robust normalisation	Median + MAD	$\mathscr {O}(N_w\,N_{\textrm{blk}})$
Stage 4: Smoothing	Gaussian convolution	$\mathscr {O}(N_w\,N_{\textrm{blk}}\,L)$

For reproducibility and implementation clarity, a complete algorithmic description of the proposed framework is provided in Appendix E.

## Real data analysis

To demonstrate the generality of the proposed framework, we apply it to two real DAS datasets representing contrasting seabed environments. The first dataset comes from the EMEC test site near Eday Island, Orkney, where Cable 4 crosses a geologically complex shallow-water region characterised by alternating bedrock, sand ripples, and erosional scarps (Here “cable 4” refers to the fourth subsea power/telemetry cable at the EMEC Eday test site, instrumented with DAS measurements in this study.) These heterogeneous conditions naturally produce repeated transitions between buried and exposed cable states. The second dataset is acquired from the OBSEA coastal observatory near Barcelona, Spain, where the cable is situated in a relatively smooth, sediment-dominated environment with more gradual variations in burial conditions.

### EMEC Eday dataset

**Fig. 2:**
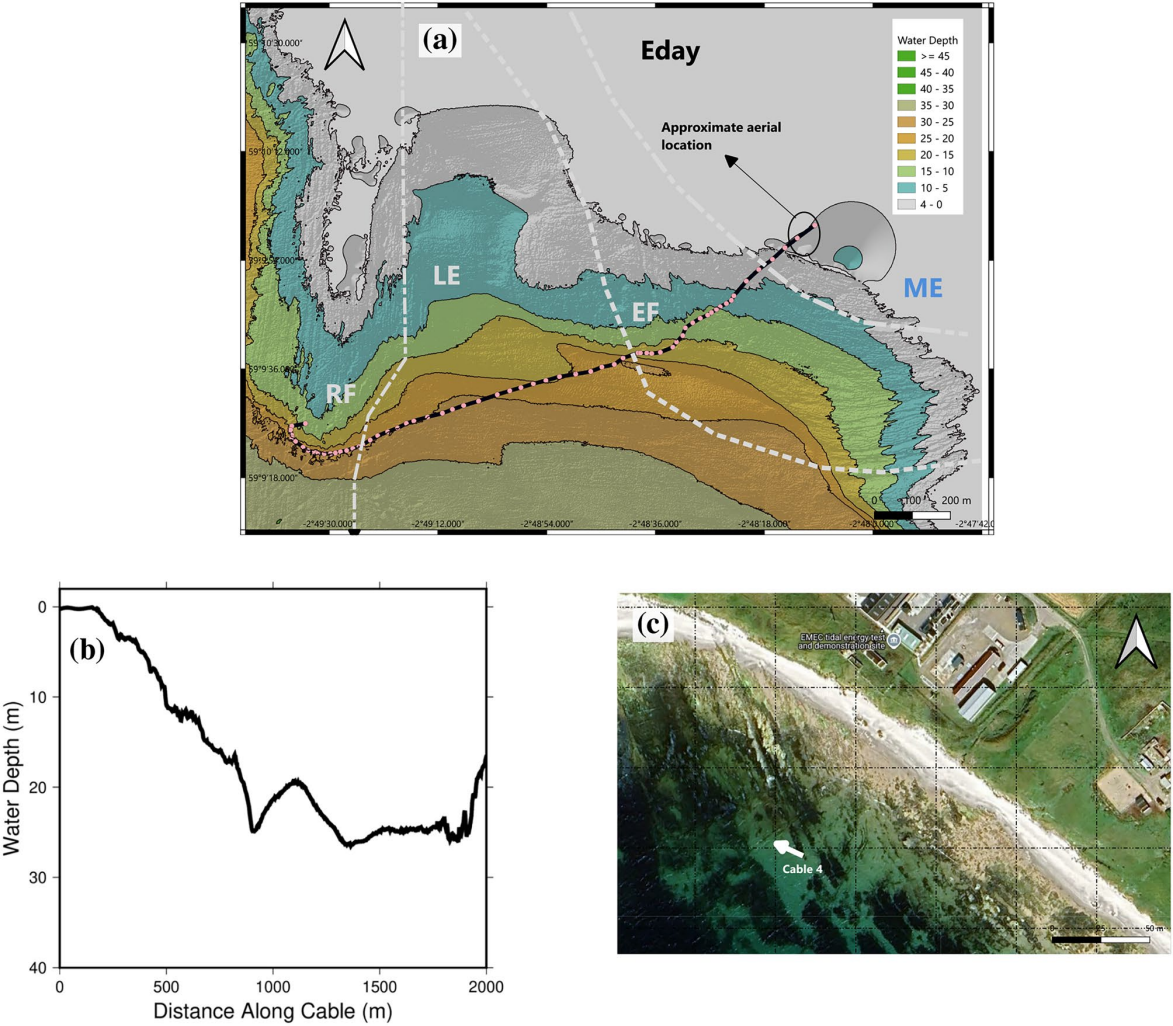
Overview of the EMEC Eday Cable 4 site. (**a**) Bathymetric map of the EMEC Eday test site, Orkney, UK, generated by the authors using UK Hydrographic Office (UKHO) bathymetric data and processed using QGIS^43,44^. The 2 km route of Cable 4 is shown by the black line (marked with pink-colored dots, used purely for cable pass contrast and distinguishability). Geological boundaries between the Rousay Flagstones (RF), Lower Eday Sandstone (LE), Eday Flagstones (EF), and Middle Eday Sandstone (ME) are indicated by dashed white-lines. The circle highlights the nearshore region shown in panel (**c**), while the inset map locates Eday within the UK. (**b**) Water-depth profile along the cable^10^. (**c**) Aerial photograph of the nearshore EMEC site. Satellite imagery was obtained from Google Earth Pro (Google LLC; Airbus imagery) and processed using QGIS^44,45^. White arrows highlight locations where the cable is directly visible on the seabed. Approximate along-cable distances are annotated for reference and correspond to the nearshore segment highlighted in panel (a).

The experimental dataset analyzed in this work originates from the european marine energy centre (EMEC) test site located off Eday Island, Orkney (UK), as shown in Fig. 2. The monitored infrastructure is a *∼*2 km shallow-water composite power cable.

The cable consists of three armoured high-voltage copper conductors and an integrated 12-core single-mode optical-fiber bundle positioned near the outer radius of the cable^9,10,21^. Its overall diameter is approximately 10 cm, with double steel-wire armouring and additional ductile-iron protection in the near-shore zone (< 15 m water depth).

As illustrated in Fig. 2, the cable is buried at the low-tide mark but lies largely exposed on the seabed elsewhere. It traverses a heterogeneous geological environment, crossing alternating zones of bedrock, sand ripples, and erosional scarps associated with the local Eday Syncline geology, including units such as the Rousay Flagstones (RF), Lower Eday Sandstone (LE), and Eday Flagstones (EF).

DAS measurements were acquired in November 2020 using a DAS system, which was connected to the onshore end of the cable and operated remotely. Data were sampled at 1 kHz with a 10 m gauge length and 2.04 m spatial channel spacing, yielding roughly 1080 channels along the entire cable^9^. During the campaign, no electrical power was transmitted, indicating that the recorded DAS response was not influenced by operational heating or electromagnetic disturbances; consequently, the burial inference relies solely on mechanically induced coupling variability.

Environmental conditions at the site are dominated by strong tidal currents (up to 4 m/s) and vigorous wave activity, providing an ideal setting to study mechanical coupling between the cable and the seabed. The resulting DAS data contains a mixture of low-frequency gravity-wave motion and high-frequency anthropogenic signals (e.g., small-vessel passages). These diverse excitations make the dataset particularly suitable for testing data-driven coupling indicators and graph-theoretic analyses.

**Fig. 3:**
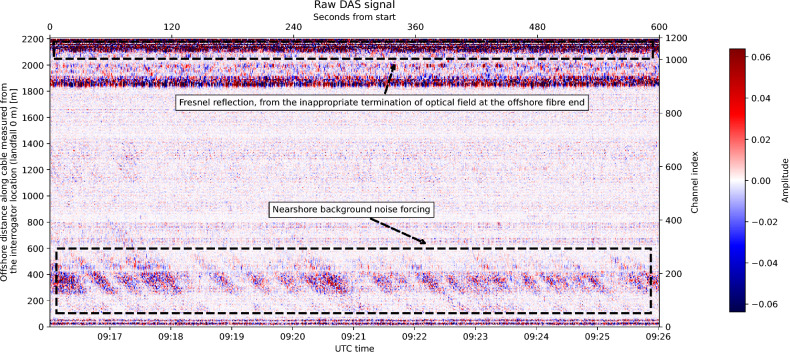
Raw DAS phase-rate data over a 600 s window. phase-rate amplitudes recorded along the 2 km EMEC Eday cable (1 kHz sampling, 2.04 m spacing, 10 m gauge length) are shown as a function of time.

Fig. 3 presents a time–distance heatmap of DAS differential phase-change measurements from the EMEC Eday dataset along the full cable length. The plot shows a 600-second snapshot of the recorded signal. Time is displayed on the horizontal axis, while the vertical axis represents distance along the cable measured from the interrogator location (landfall at 0 m). Darker colours indicate higher differential phase-change amplitudes.

The figure highlights the complex spatio-temporal structure of the DAS (differential phase-rate) measurements. A rectangular region between approximately 200–600 m reveals coherent oblique streaks associated with environmental forcing. This behaviour is consistent with persistent nearshore excitation, driven by shallow-water processes and local bathymetric variability. In particular, the depth transition observed in the panel (b) of Fig. 2 (approximately 200–400 m) suggests the presence of rocky features and partial cable spanning, which increase susceptibility to wave-induced and flow-induced forcing. These effects contribute to elevated amplitudes and band-limited energy near the interrogator end. Additional variability in this region may arise from installation-related factors, such as trench transitions or heterogeneous sediment cover, leading to increased spatial irregularity in cable coupling.

A second annotated region near the offshore end of the cable exhibits strong, persistent high-amplitude signals. These are attributed to Fresnel reflections caused by imperfect optical termination at the distal fibre end, rather than physical environmental forcing.

Overall, the nearshore section (lower part of the plot) is characterised by a persistently elevated baseline amplitude due to strong shallow-water forcing, including surface gravity waves, wave–current interactions, and shoreline reflections. In contrast, deeper offshore sections (upper part of the plot) show a lower baseline amplitude, with intermittent high-amplitude bursts likely associated with transient localised excitations, such as interactions near the tidal turbine monopile where the cable may be more exposed.

This behaviour is also evident in the raw DAS phase-rate signal and is further illustrated in Appendix C, where a one-dimensional RMS amplitude profile is provided. While the RMS representation improves interpretability, the raw heatmap clearly demonstrates the highly complex, non-stationary, and noise-dominated nature of DAS measurements at high sampling rates.

Direct analysis of such raw data is computationally demanding and does not provide a clear indication of burial state. This motivates the use of advanced signal-processing approaches that incorporate physical insight, such as spectral fingerprint construction and coherence-based analysis, introduced in the following sections.

#### Results for EMEC Eday

**Fig. 4:**
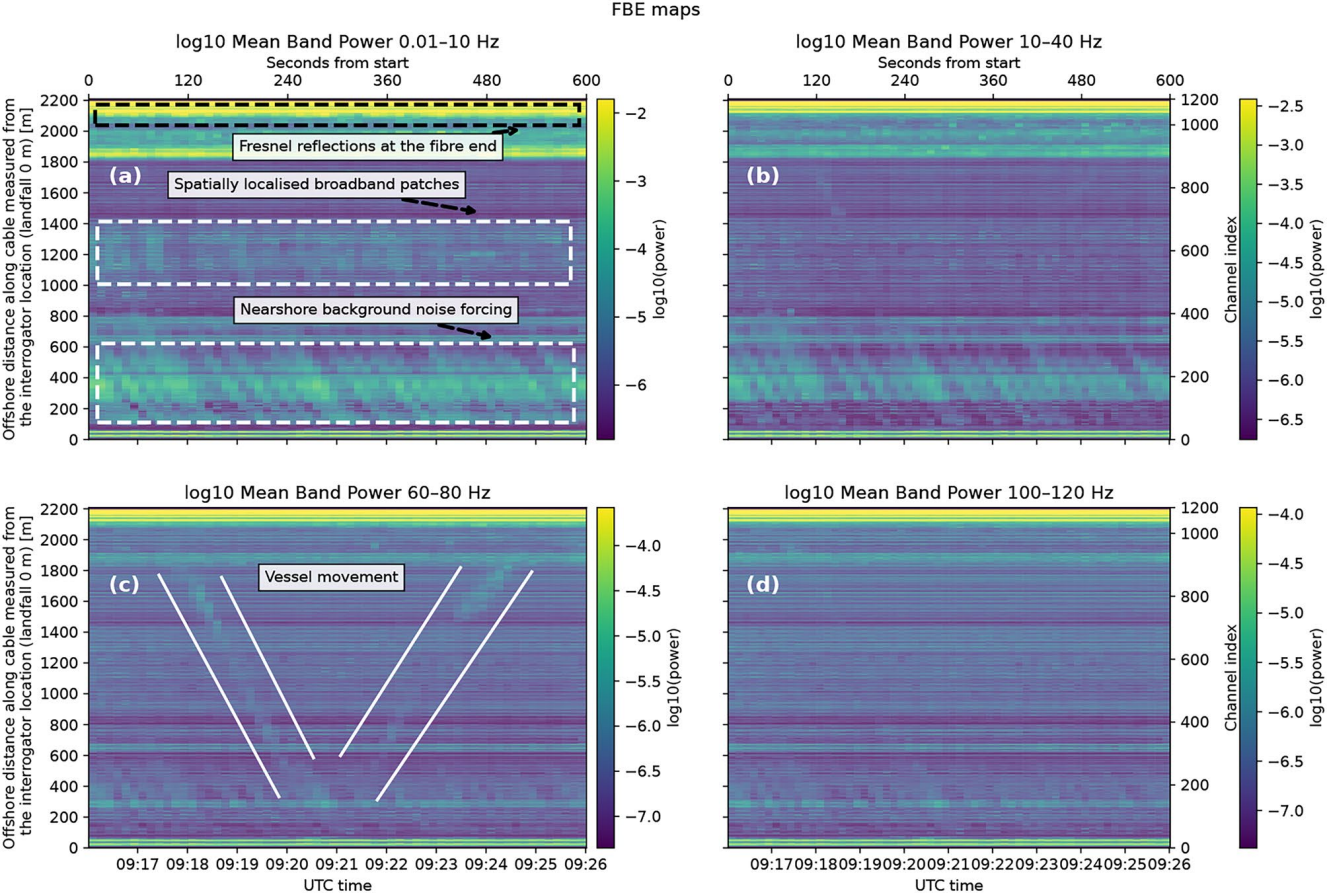
Time–distance maps of FBE for the EMEC Eday dataset, defined as the band-integrated spectral power obtained by summing the squared Fourier amplitudes (periodogram power; see Appendix A) over the selected frequency interval for each channel and time window (see Methods). Values are displayed in $\log _{10}$ scale for clarity. Panels show (**a**) shows spatially localised low-frequency broadband patches in mid-offshore region interpreted as prospective current/turbulence-driven forcing together with continuous elevated nearshore arrivals, consistent with shallow-forcing modulations over 0.01–10 Hz, (**b**) nearshore forcing modulation seen over 10–40 Hz, (**c**) highlights coherent vessel move-outs in 60–80 Hz, and (**d**) 100–120 Hz.

The FBE maps for the EMEC Eday dataset illustrate how the band-limited spectral content of the DAS differential phase field varies with time and distance along the approximately 2 km cable (Fig. 4). The FBE is computed as the band-integrated spectral power, obtained by summing the squared Fourier amplitudes (periodogram power) within a selected frequency band for each channel and time window. This representation provides a compact description of how the variance of the DAS response is distributed across physically meaningful frequency ranges (see Section "Spectral fingerprint construction" and Appendix A). Additional time–frequency representations across the dataset, further supporting the selected frequency bands, are provided in Appendix D.

Each panel shows the $\log _{10}$ mean spectral power within a specific frequency band, enabling separation of low-frequency hydrodynamic forcing from higher-frequency environmental and anthropogenic excitations.

Panels (a) and (b) in Fig. 4, corresponding to the 0.01–10 Hz and 10–40 Hz bands, are dominated by low-frequency processes associated with surface gravity waves, tidal flow, and wave–current interactions. A persistent region of elevated energy is observed in the nearshore section (approximately 200–400 m), appearing as a continuous elevated band energy over nearshore channels. This feature is consistent with the coherent structures observed in the raw DAS phase data (Fig. 3) and reflects sustained shallow-water forcing modulated by local bathymetric variability. In particular, the depth transition shown in Fig. 3 suggests the presence of rocky features and partial cable spanning, which increase sensitivity to environmental excitation. Additional horizontal structures at intermediate distances indicate spatially fixed variations in mechanical coupling and environmental loading. While these features remain visible in panel (b), their amplitudes are reduced, reflecting attenuation and redistribution of energy into secondary interactions.

A second prominent feature appears near the offshore end of the cable as a region of persistently elevated spectral power. This behaviour is primarily attributed to Fresnel reflections arising from imperfect optical termination at the distal fibre end, where termination is uncontrolled and located underwater. A similar, though weaker, reflection is also observed near the interrogator (landfall, 0 m), indicating that Fresnel effects are present at both ends of the fibre. The stronger response at the offshore end is consistent with poorer termination conditions, whereas differences observed at the interrogator side (particularly between campaigns studied here) are likely related to variations in launch connector conditions. The presence of this feature across all frequency bands, as well as in the raw DAS phase data (Fig. 3), confirms its origin as an acquisition-related boundary effect rather than a physical environmental process.

Spatially localised broadband patches observed between approximately 1000–1400 m (with representative activity around *∼*1200 m) do not show a consistent propagation slope and are confined to limited channel ranges. Such non-propagating, intermittent amplitude enhancements are consistent with spatially heterogeneous near-bottom forcing, such as current-driven turbulence, flow separation, or intermittent cable–seafloor contact. These processes can induce intermittent cable vibrations and spatially heterogeneous forcing along the cable, in agreement with previous studies^46,47^.

Panel (c) in Fig. 4, corresponding to the 60–80 Hz band, highlights vessel-passage induced excitation. Dashed lines trace oblique moveout patterns associated with propagating sources along the cable, providing clear evidence of vessel motion. In this frequency range, vessel-related acoustic–mechanical energy is enhanced and better separated from lower-frequency background forcing, allowing improved visualisation of moving sources.

Panel (d) in Fig. 4, covering the 100–120 Hz band, exhibits low spectral power and limited spatial organisation. Beyond the nearshore region, the signal appears largely noise-like due to strong high-frequency attenuation, gauge-length averaging, and DAS fading effects, rather than the absence of physical forcing.

Overall, the FBE maps reveal spatially coherent low-frequency features that are largely fixed in distance, reflecting persistent environmental forcing and coupling conditions, while higher-frequency bands show reduced coherence and visibility due to attenuation and measurement limitations. While the FBE representation effectively captures the distribution of spectral energy, it does not directly resolve burial state, motivating the need for coherence-based analysis introduced in the following sections.

It is also important to note that Fig. 3 provides a broadband time–distance representation of the raw DAS response, capturing overall forcing variability and coherent arrivals. In contrast, Fig. 4 complements this view by isolating frequency-specific contributions through FBE mapping, enabling clearer discrimination between persistent low-frequency ocean forcing and intermittent higher-frequency excitation, such as vessel-passage induced or cable-related mechanical vibration (e.g., current-driven strumming or vortex-induced vibration), which is more sensitive to the cable–seabed coupling state.

**Fig. 5:**
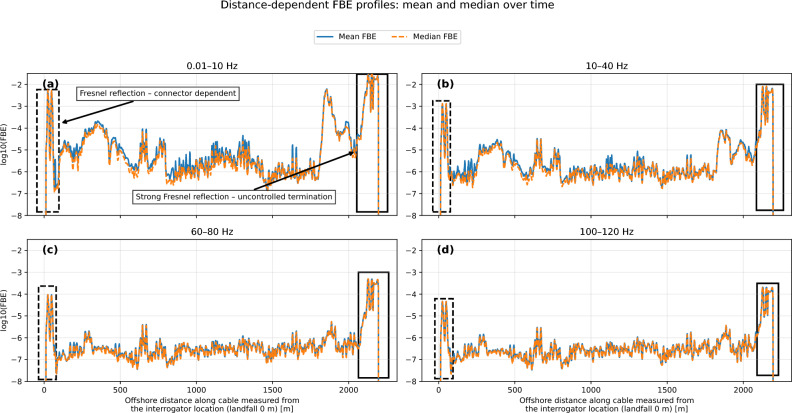
Distance-dependent profiles of FBE for the EMEC Eday dataset. Solid and dashed curves denote the mean and median FBE over time windows (600 seconds). FBE represents band-integrated spectral power (see Section "Spectral fingerprint construction" and Appendix B).

The distance-dependent FBE profiles (Fig. 5) provide a compact summary of the dominant spatial patterns observed in the time–distance heatmaps. While the full FBE maps capture detailed temporal variability, they can obscure persistent spatial trends relevant to cable–seafloor coupling. To address this, the FBE is averaged over all time windows to obtain mean profiles, and complemented by median profiles as a robust alternative that reduces sensitivity to transient high-amplitude events such as vessel passages.

These distance profiles emphasise stable spatial gradients and transition zones along the cable more clearly than the full time–distance representation. In particular, regions of consistently elevated or reduced spectral power become more apparent, allowing clearer identification of spatial variations in coupling conditions.

The profiles also retain signatures associated with Fresnel reflections, which are explicitly indicated in Fig. 5 using two types of annotations. Dashed rectangular regions correspond to Fresnel reflections near the interrogator (landfall, 0 m), where the response is influenced by connector-dependent conditions and may vary between campaigns. Solid rectangular regions mark stronger Fresnel reflections near the offshore end of the cable, arising from uncontrolled termination of the optical field. The distinction between these two regimes highlights the different physical origins of the observed boundary effects and ensures that they are not misinterpreted as environmental or coupling-related features.

Overall, the distance-dependent FBE profiles provide a clearer view of persistent spatial structure along the cable, complementing the time–distance maps while suppressing transient variability. Although these profiles effectively summarise the spatial distribution of spectral energy, they do not directly resolve burial state, reinforcing the need for coherence-based analysis introduced in the following sections.

**Fig. 6:**
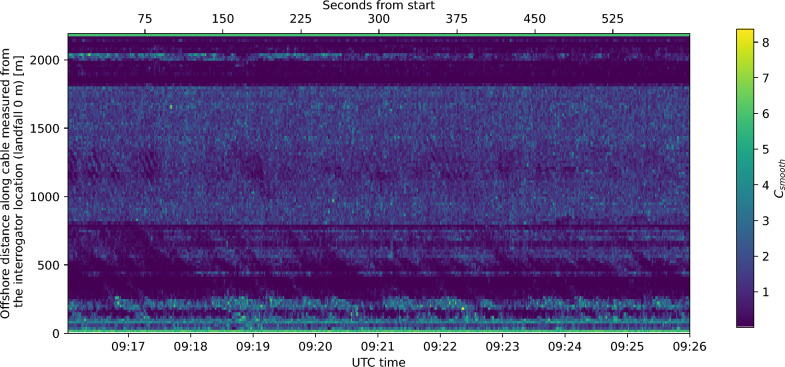
Smoothed and normalised Poincaré–style coherence map $C_{\textrm{smooth}}$ derived from spectral fingerprints, highlighting persistent spatial patterns in relative spectral variability along the cable.

Fig. 6 shows the smoothed, normalised Poincaré-style coherence map $C_{\textrm{smooth}}$, derived from spectral fingerprints applied to the EMEC Eday dataset (see Eq. (24)). Compared to the raw FBE maps, this coherence-based representation suppresses channel-scale noise and enhances spatially persistent patterns by emphasising relative spectral variability between neighbouring channels rather than absolute energy levels. Regions of elevated $C_{\textrm{smooth}}$ correspond to sections where spectral fingerprints vary more irregularly across space, consistent with dynamically less constrained cable behaviour, while lower values indicate smoother and more spatially coherent motion along the fiber.

**Fig. 7:**
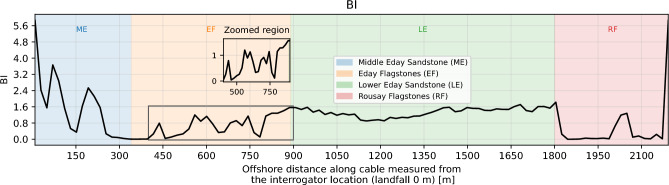
BI (interpreted as a coupling-state indicator) for the EMEC Eday dataset. The zoomed region highlights the Eday Flagstones (EF) zone (400–750 m from the shore), where the BI shows a reduced overall level compared to the preceding section, with noticeable spatial variability, consistent with geological boundaries between the Rousay Flagstones (RF), Lower Eday Sandstone (LE), Eday Flagstones (EF), and Middle Eday Sandstone (ME).

The time–median of $C_{\textrm{smooth}}$ profile shown in Fig. 7 provides a one–dimensional geological indicator along the interrogated fibre–optic cable (Cable–4) at the EMEC test site, Eday. This profile is obtained by Eq. (26), derived from the normalised Poincar’e–style coherence map, thereby emphasising persistent spatial variability while suppressing transient fluctuations. The resulting profile should be interpreted in conjunction with the environmental context presented in Fig. 2, which provides the corresponding bathymetric and geological setting along the cable route.

To aid interpretation, Fig. 7 also includes mapped lithological unit boundaries along the cable route, including the Rousay Flagstones (RF), Lower Eday Sandstone (LE), Eday Flagstones (EF), and Middle Eday Sandstone (ME). These boundaries provide an independent sedimentological framework for interpreting coupling transitions, since seabed stiffness, sediment availability, and exposure likelihood can vary strongly across these units. We emphasise that these geological boundaries do not constitute direct ground-truth burial measurements; rather, they provide contextual constraints that help explain persistent changes in the inferred coupling metric.

The BI derived from the Poincaré spectral coherence formulation is interpreted as a coupling irregularity indicator: buried or well-supported segments are expected to behave more uniformly across adjacent channels due to sediment confinement and damping, producing low BI values. In contrast, exposed or weakly supported segments can experience spatially heterogeneous forcing and free-span dynamics, resulting in elevated fingerprint irregularity and higher BI values.

Fig. 7 shows that the inferred BI exhibits distinct spatial structure that aligns with mapped lithological units along the cable route. In the nearshore section (0–300 m), BI values show elevated variability. The cable is protected by ductile–iron shells and subjected to strong hydrodynamic forcing, resulting in BI irregularity in consonance with enhanced motion irregularity due to complex boundary conditions and energetic shallow-water dynamics, consistent with stronger shallow-water forcing and installation-related coupling heterogeneity near the landfall. Beyond this region, the BI decreases and remains persistently low across the interval approximately 200–350 m, suggesting a buried or well-supported segment.

In addition to the nearshore variability, the BI profile contains multiple offshore peaks and troughs, e.g., 450–900 m in the Eday Flagstones (EF) region, characterised by rock scarps. Correspondingly, the BI value variability is indicative of weakly supported or perhaps even free-span segments of the cable. More consistent BI zones occur within the LE (elevated but consistent between 1400-1750 m) and RF (reduced on either side of the broad peak between 1950-2100 m) intervals, indicative of well supported but with reduced mechanical constraint and buried cable sections, respectively. This spatial trend, is consistent with the findings of Harmon et al^10^., who identified over the same interval (i.e., the elevated but consistent region) as likely partially buried based on vessel-induced frequency-band energy signatures.

Notably, elevated BI values tend to occur near mapped lithological transitions such as LE-RF and EF-LE, where abrupt changes in seabed material and stiffness may produce sharp coupling contrasts. In contrast, the ME-EF transition appears more gradual in both the geological interpretation and the BI response, which may indicate that coupling conditions evolve progressively in this region offshore. This could reflect spatially distributed sediment transport, scour, and partial sediment cover that smooths the effective mechanical contrast experienced by the cable, producing a gradual rather than step-like coupling transition. These interpretations are based on the relative BI magnitude and its spatial persistence, and are supported qualitatively by geological mapping and independent observations. We note that the lithological overlay is not direct burial ground truth, but provides contextual constraints consistent with the expectation that seabed stiffness and sediment availability modulate cable coupling, which is no surprise at this high-tidally active site.

This visual comparison highlights that elevated BI values align with intervals of reduced sediment cover and/or lithological transitions, supporting the interpretation that the Poincaré coherence metric captures coupling changes associated with burial state.

### OBSEA dataset

**Fig. 8:**
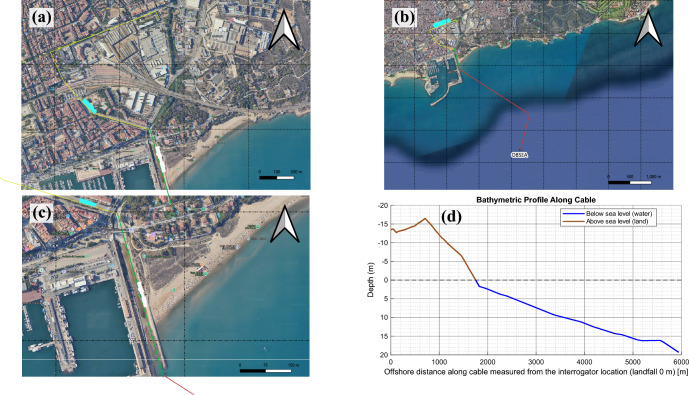
Figure 8. Overview of the OBSEA cable layout and bathymetric context used in this study. (**a**) Onshore terrestrial section of the OBSEA cable from the Ground Station to the beach manhole. (**b**) Offshore cable route from the coastline to the OBSEA seafloor observatory. (**c**) Zoomed-in view of the nearshore transition between the terrestrial and marine cable sections. Satellite imagery in panels (**a**)–(**c**) was obtained from Google Earth Pro (Google LLC; Airbus imagery) and processed using QGIS^44,48^. (**d**) Bathymetric profile showing depth variation along the cable route. In subfigures (**a**), (**b**), and (**c**), the yellow, green, and red lines indicate the approximate cable route corresponding to the onshore terrestrial section, the nearshore transition zone between the terrestrial and marine environments, and the offshore section, respectively.

The second DAS dataset analysed in this study was acquired at the OBSEA cabled observatory, offshore Barcelona (see fig.8). DAS measurements were acquired using a sampling rate of 2000 Hz, a gauge length of 20 m, and a spatial channel spacing of approximately 1.275 m

It comprised controlled multi-day field experiments staged around 5.8 km section of a telecommunication-power cable, interrogated using an advanced version of the system used in the campaign discussed above, where the advancement comprised several optoelectronic upgrades that supported seamless remote operability. The interrogation comprised both nearshore shallow regions and deeper offshore segments, thereby enabling a diverse range of seabed–cable interaction states. A comprehensive experimental plan comprised baseline recordings, repeated vessel transects, relative to the cable, tests with frogmen/divers, impulsive sources, and several long-duration passive recordings for microseismic and ambient noise monitoring. Field observations by frogmen/divers and vessel operators confirmed cables’ strong spatial heterogeneity in burial state, with transitions between unburied, partially buried, and deeply buried sections. It therefore positions the OBSEA-dataset as a unique real-world benchmark for evaluating FBE- and Poincare spectral coherence-based burial detection, as it combines well-documented geometry, controlled offsets, and naturally varying seabed contact conditions over kilometres scale.

**Fig. 9:**
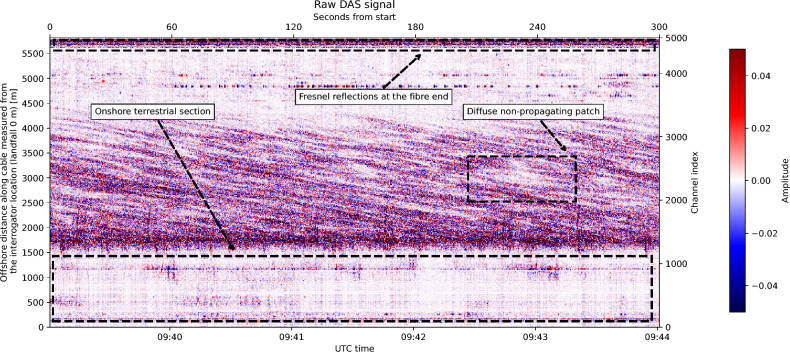
Raw DAS differential change in phase rate data over a 300-s window for OBSEA.

The heatmap generated from DAS data for the OBSEA cable in Fig. 9 shows a 300-second snapshot of the differential phase-rate field along the full 5.8 km cable. The plot displays the linear amplitude, with time on the horizontal axis and distance along the cable (0 m at landfall) on the vertical axis. As expected for high-sampling-rate DAS acquisition (e.g., 2 kHz in this campaign), the recordings capture strong broadband ambient motion and spatially heterogeneous forcing from environmental and anthropogenic sources. These effects can obscure burial or exposure signatures in raw t–x plots, motivating the use of dedicated diagnostics such as spectral analysis and coherence-based metrics.

A pronounced high-amplitude band is observed near the offshore end of the cable, corresponding to Fresnel reflections arising from imperfect termination of the optical field at the fibre boundary (see top annotation in Fig. 9). This feature is an instrumental artefact and should not be interpreted as a physical environmental process.

Near the shoreward (e.g., terrestrial) end of the array (up to 1.7 km), signal amplitudes indicate coherent propagating arrivals, consistent with land-based DAS observations indicative of traffic, cultural, and near-surface seismic noise-fields. Moving offshore, the signal exhibits diffused and spatially heterogeneous forcing (see, e.g^46,47^.,), with intermittent broadband patches (e.g., around 3000 m offshore distance at UTC 09:43). These diffused non-propagating patches can be caused due to turbulence-driven excitation and/or near-bottom variability including hydrodynamic excitation, intermittent sediment contact, seabed roughness effects etc., that obscure direct visual identification of burial or exposure states in the raw t–x plot.

Furthermore, the presence of coherent obliquely propagating arrivals indicates wave-like forcing, as also observed in Fig. 9, which exhibits apparent propagation velocities on the order of several metres per second, consistent with ocean-surface-gravity-wave-related (OSGW) forcing. While OSGW energy is typically concentrated in the sub-Hz band (approximately 0.01–1 Hz), interactions between ocean-wave forcing and the seabed can generate higher-frequency seismo-acoustic responses, including interface-wave energy (e.g., Scholte-type modes) and other mechanically coupled motions along the seafloor–water interface. Such responses may become more visible in weakly coupled or exposed cable segments. In this study, spectral fingerprints are constructed using representative low- and high-frequency bands (0.01–10 Hz, 10–40 Hz, 60–80 Hz, and 100–120 Hz) selected to capture contrasting components of the measured DAS response. Similar observations have been reported in seafloor DAS studies of ocean–solid Earth interactions^46^ and in recent work attributing enhanced higher-frequency OSGW signatures to coupling variability and bathymetric complexity^47^.

Overall, the combination of strong environmental loading, channel-to-channel variability due to residual phase fading, frequency-dependent coupling, and instrumental artefacts produces broadband motion that complicates direct identification of burial-related signatures in short-duration snapshots, motivating the use of more robust, spatially informed diagnostics.

In addition, a one-dimensional RMS amplitude profile computed from the raw DAS phase-rate signal is provided in Appendix C. This representation offers a complementary, distance-dependent view of the signal strength, confirming the dominant spatial patterns observed in the heatmaps while providing a quick and intuitive summary of amplitude variations along the cable. As such, it serves as a useful supplementary diagnostic to support the interpretation of the main figures.

#### Results for OBSEA dataset

**Fig. 10:**
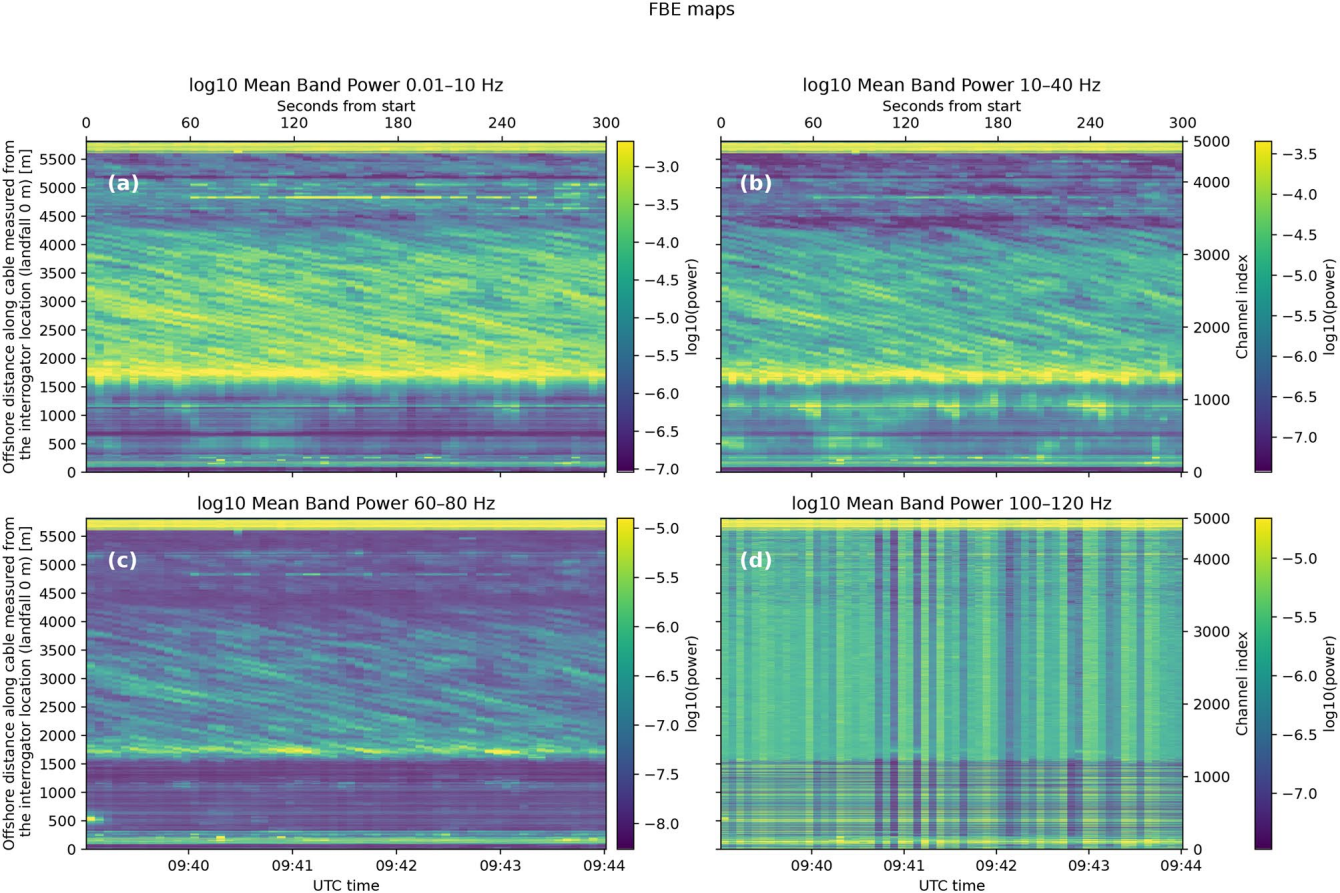
Time–distance maps of FBE for the OBSEA dataset, defined as the band-integrated spectral power obtained by summing the squared Fourier amplitudes (periodogram power; see Appendix A, Eq. 31) over the selected frequency interval for each channel and time window (see Methods). Values are displayed in $\log _{10}$ scale for clarity. Panels show (**a**) 0.01–10 Hz, (**b**) 10–40 Hz, (**c**) 60–80 Hz, and (**d**) 100–120 Hz.

The FBE maps for the OBSEA dataset illustrate how the spectral content of the DAS data varies with time and distance along the approximately 5.8 km cable (Fig. 10). Each panel displays the $\log _{10}$ mean spectral power within a specific frequency band, enabling separation of low-frequency hydrodynamic forcing from higher-frequency environmental and anthropogenic excitations.

Panels (a) and (b) in Fig. 10, corresponding to the 0.01–10 Hz and 10–40 Hz bands, exhibit behaviour consistent with the dominant low- and mid-frequency responses observed in the OBSEA dataset. A broad region between approximately 1500 and 4000 m shows elevated spectral energy associated with coherent propagating arrivals, indicative of wave-like forcing^46,47^. The feature is most prominent in the lowest-frequency band and persists with reduced amplitude in the 10–40 Hz band, reflecting attenuation and redistribution of energy into secondary mechanical and hydrodynamic interactions.

In the 60–80 Hz band shown in panel (c) in Fig. 10, the same spatial region exhibits only a weak enhancement in spectral power relative to the surrounding cable. The reduced amplitudes are consistent with strong sediment damping and limited sensitivity to mid-frequency excitation in this sediment-dominated environment. No clear vessel-related signatures are observed within this time window, suggesting limited vessel activity or insufficient signal strength for detection in this frequency range.

Panel (d) in Fig. 10, covering the 100–120 Hz band, is characterised by very low spectral power and limited spatial coherence along the cable. The broad low-frequency structure observed between 1500 and 4200 m is largely suppressed at these higher frequencies. The remaining signal is dominated by weak, spatially incoherent and temporally intermittent patterns, consistent with strong high-frequency attenuation, gauge-length averaging, and DAS fading effects rather than organised environmental forcing.

A region near the offshore end of the cable exhibits persistently elevated spectral power, attributed to Fresnel reflections arising from imperfect optical termination at the fibre boundary. This feature is clearly visible across all panels in Fig. 10, consistent with the broadband nature of the effect. In contrast to the EMEC dataset, the corresponding near-interrogator Fresnel response in the OBSEA data is weak and not clearly distinguishable, likely reflecting differences in connector conditions between campaigns, as also noted previously. The consistency of this offshore feature with the raw DAS observations (Fig. 9) confirms its origin as an acquisition-related boundary effect rather than environmental forcing.

Overall, the FBE maps highlight physically meaningful frequency-dependent structure: the lowest-frequency band (0.01–10 Hz) exhibits persistent energy consistent with background ocean forcing, whereas higher-frequency bands (60–80 Hz and 100–120 Hz) show more intermittent excitation patterns that are expected to be more sensitive to local coupling conditions and episodic anthropogenic activity. As in the EMEC analysis, these FBE representations do not directly resolve burial state but provide the spectral context required for constructing the fingerprint-based coherence maps presented in the following sections.

**Fig. 11:**
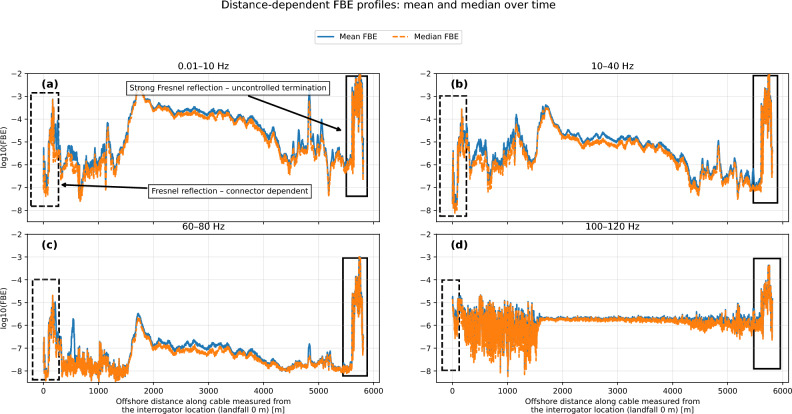
Distance-dependent profiles of FBE for the OBSEA dataset. Solid and dashed curves denote the mean and median FBE over time windows (300 seconds). FBE represents band-integrated spectral power (see Section "Spectral fingerprint construction" and Appendix B).

To complement the time–distance FBE maps for the OBSEA dataset, distance-dependent profiles are derived by aggregating the spectral energy over time (Fig. 11). While the heatmaps capture detailed temporal variability, these profiles provide a compact representation of the spatial distribution of band-limited energy along the cable.

Specifically, the mean FBE is computed across all time windows to characterise the overall energy distribution, while the median FBE is used as a robust alternative that reduces sensitivity to transient high-amplitude events such as vessel passages. This dual representation enhances the visibility of persistent spatial gradients and transition zones, enabling clearer interpretation of coupling variability along the cable.

The profiles also retain signatures associated with Fresnel reflections, which are indicated using the same annotation scheme as in the EMEC dataset. Dashed rectangular regions correspond to Fresnel reflections near the interrogator (landfall, 0 m), associated with connector-dependent boundary conditions. In the OBSEA dataset, this near-end response is comparatively weak and not strongly expressed. In contrast, solid rectangular regions mark strong Fresnel reflections near the offshore end of the cable, arising from uncontrolled optical termination. This consistent annotation highlights the shared physical origin of boundary effects across datasets while capturing their differing relative magnitudes.

For consistency with the spectral analysis framework, the FBE shown here corresponds to band-integrated spectral power, obtained by integrating the PSD over the selected frequency intervals (see Section "Spectral fingerprint construction" and Appendix A).

**Fig. 12:**
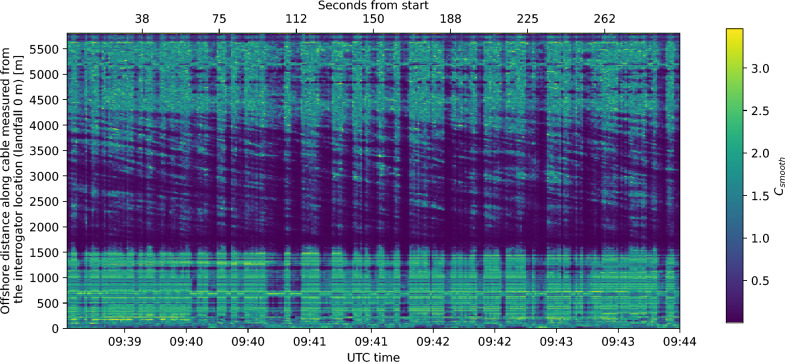
Smoothed and normalised Poincaré–style coherence map $C_{\textrm{smooth}}$ derived from spectral fingerprints, highlighting persistent spatial patterns in relative spectral variability along the cable.

Fig.12 shows the smoothed and normalised Poincaré-style coherence map $C_{\textrm{smooth}}$ derived from spectral fingerprints. Compared to the raw FBE maps, this representation suppresses channel-scale noise and emphasises persistent spatial variability in relative spectral structure rather than absolute energy. A spatially extensive region of consistently low $C_{\textrm{smooth}}$ values is clearly visible along the central portion of the cable, appearing as a coherent low-value (blue) band that persists throughout the analysed time window. This indicates spatially smooth and internally consistent spectral behaviour across neighbouring channels in this section. In contrast, regions with elevated $C_{\textrm{smooth}}$ exhibit greater spatial variability, reflecting more irregular spectral differences between adjacent channels.

Overall, the OBSEA results demonstrate how the coherence-based representation complements energy-based spectral maps by extracting stable, large-scale spatial patterns from noisy DAS measurements. While the FBE maps primarily reflect environmental forcing and attenuation effects, the Poincaré coherence map provides an additional layer of information on spatial variability that is not readily discernible from spectral energy alone.

**Fig. 13:**
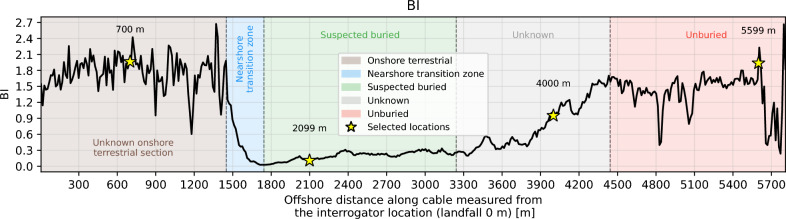
BI along the OBSEA cable. The coloured segments denote burial conditions reported from diver-based inspection surveys, including suspected buried, unknown, and unburied regions. The BI profile highlights spatial variations in coupling behaviour that align with these independently reported burial states. The stars indicate the selected locations presented in Fig. 14.

The BI is presented in Fig. 13 reveals a set of distinct spatial regimes along the OBSEA cable that are consistent with independently reported burial states from diver and vessel observations.

From the interrogator on shore to approximately 1743.5 m (the point at which the cable is reported to go offshore), the BI exhibits persistently elevated and locally fluctuating values. This behaviour indicates increased spatial variability in the spectral response and is consistent with dynamically irregular motion expected for an exposed or weakly supported cable under spatially heterogeneous environmental forcing.

From approximately 1743.5 to 3300 m, the BI values decrease to relatively low levels, indicating increased spatial coherence in the spectral response. This behaviour reflects a progressive increase in mechanical constraint as the cable becomes more strongly coupled to the surrounding sediment, consistent with the interval classified as “suspected buried” (1743.5–3242.9 m) based on comments and observations from vessel crew and frogman/divers.

Between approximately 3300 and 4300 m, the BI gradually increases with relatively small fluctuations, indicating a moderate reduction in spatial coherence compared to the preceding buried segment. This region corresponds to the “unknown” classification (3242.9–4342.3 m), where no direct burial information is available from field observations by vessel crew and frogmen/divers. While the BI suggests relatively coherent behaviour in this interval, no definitive interpretation is made due to the absence of independent observational constraints.

Beyond approximately 4445 m toward the offshore end, the BI increases and exhibits stronger fluctuations, indicating a return to spatially irregular motion typical of exposed or weakly supported cable segments. This behaviour is consistent with the interval classified as “unburied” (4442.5 m to the offshore end) based on field observations from vessel crew and frogmen/divers.

Overall, the time-median BI provides a stable and physically interpretable indicator of large-scale variations in cable–seabed coupling along the OBSEA route. The strong agreement with the annotated burial-state regions in Fig. 13 demonstrates the capability of the proposed coherence-based framework to resolve buried, transitional, and exposed cable segments under realistic shallow-water conditions.

## Discussion

The results from both EMEC Eday and OBSEA demonstrate that the proposed Poincaré spectral-coherence framework reliably identifies spatial transitions in cable–seafloor coupling across diverse oceanographic conditions.

**Fig. 14:**
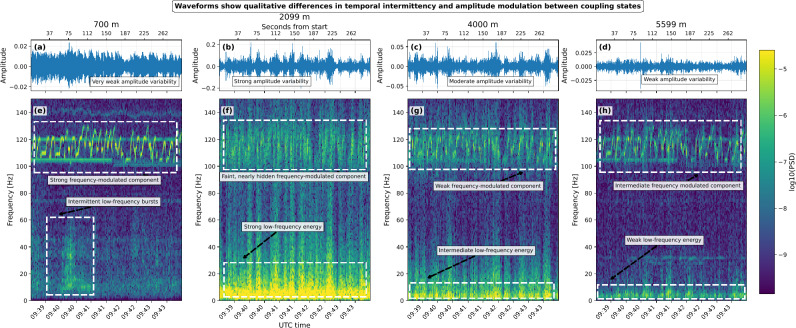
Raw DAS time series and corresponding spectrograms from four OBSEA channels located at approximately 700 m, 2100 m, 4000 m, and 5600 m, selected to represent distinct coupling regimes along the cable. These distances are defined with respect to the offshore distance along the cable measured from the interrogator location (landfall at 0 m), and are marked with star symbols in Fig. 13. Subplots (**a**)–(**d**) show the raw signal waveforms, illustrating qualitative differences in temporal intermittency and amplitude variability between coupling states, while (**e**)–(**h**) present the corresponding spectrograms, highlighting frequency-dependent behaviour across these locations.

In the OBSEA dataset, Fig. 14 presents representative raw DAS-data time series and corresponding spectrograms from four along-cable locations (700 m, 2099 m, 4000 m, and 5599 m). These distances are defined with respect to the offshore distance along the cable measured from the interrogator location (landfall at 0 m) and are marked with star symbols in Fig. 13. The selected locations span distinct coupling regimes, including the onshore (terrestrial) section (700 m), a suspected buried mid-section (2099 m), an intermediate transition zone (4000 m), and a shallow offshore section (5599 m).

The waveform panels highlight clear differences in temporal intermittency and amplitude variability across these coupling conditions, while the spectrograms reveal how energy is distributed across frequency bands.

High-frequency (100–140 Hz) amplitude modulation is clearly observed in the onshore location (700 m), where strong and intermittent amplitude fluctuations are present. In contrast, this high-frequency content is significantly attenuated in the transition region (4000 m) and becomes nearly absent in the buried segment (2099 m), which corresponds to the lowest BI values in Fig. 13

In the offshore section (5599 m), associated with relatively higher BI values, frequency-modulated components reappear but remain weaker than those observed in the onshore region. However, these components are more clearly defined than in the transition and buried regions, reflecting differences in coupling conditions.

Energy observed within the 100–140 Hz band reflects intermittent high-frequency excitation and broadband mechanical disturbances affecting the cable. Although the precise forcing mechanisms are not identified here, such components are generally more strongly attenuated by sediment damping and cable–seafloor coupling than lower-frequency motions. Consequently, this frequency range provides additional sensitivity to spatial variations in coupling state and contributes to discrimination between regions exhibiting differing mechanical interaction with the seabed.

At low frequencies (<30 Hz), strong and persistent energy is observed in the buried section (2099 m), consistent with background ocean forcing acting on well-coupled cable segments. This low-frequency energy is reduced in the offshore region (4000–5099 m), reflecting changes in coupling conditions and environmental forcing. In contrast, the onshore (terrestrial) location (700 m) does not exhibit comparable low-frequency energy, as it is not influenced by nearshore hydrodynamic forcing.

The waveform amplitudes further support these observations, highlighting distinct amplitude characteristics between terrestrial, buried, and offshore sections. This demonstrates the importance of spatially varying cable–environment coupling in shaping the DAS response.

Overall, these observations demonstrate the critical role of cable–seabed coupling in controlling the measured DAS signal characteristics, both in the time and frequency domains. Understanding this spatial variability is essential for correctly interpreting sensed phenomena.

Finally, intermittent low-frequency bursts observed in the onshore spectrogram may be associated with anthropogenic activity (e.g., nearby railway crossings) rather than ocean-driven processes, although a detailed investigation of this effect is beyond the scope of the present study.

Burial can reduce sensitivity to certain high-frequency water-column due to sediment damping, but it may simultaneously improve coupling to solid-earth seismic wavefields, potentially enhancing the detectability of regional and teleseismic phases depending on frequency content and local sediment properties^39^. Conversely, exposed or suspended sections often show enhanced responsiveness to hydrodynamic and anthropogenic forcing, but may suffer from elevated broadband noise and resonant artefacts that complicate interpretation. These effects illustrate how spatial variations in coupling conditions–primarily driven by burial–reshape environmental and anthropogenic signatures along the cable.

Bathymetry may influence the inferred coupling metric $C_{\textrm{eff}}$ both indirectly through burial persistence and directly through the forcing environment. Water depth modifies the dominant excitation regime experienced by the cable: surface gravity wave orbital motions decay with depth, reducing direct near-bed wave forcing offshore, while deeper-water segments may be increasingly influenced by background currents, internal wave activity, and seismo-acoustic coupling mechanisms. Bathymetric gradients may additionally influence sediment transport pathways and deposition, thereby controlling the likelihood of persistent burial or exposure. Consequently, spatial trends in $C_{\textrm{eff}}$ may reflect a combination of burial-driven coupling changes and depth-dependent variations in ambient forcing. This further motivates interpreting the proposed BI primarily as a cable–seafloor coupling indicator, with burial state as a dominant but not exclusive control.

In addition to water depth, bathymetric rugosity (i.e., small-scale roughness and relief) may influence the inferred coupling metric $C_{\textrm{eff}}$ through mechanisms not directly related to burial. Rough seafloor topography can intensify near-bottom turbulence via flow separation and eddy formation, generating spatially heterogeneous forcing that increases channel-to-channel spectral variability along the cable. Rugosity may also affect the observed DAS field through scattering and mode conversion of seismo-acoustic surface waves, potentially modifying the spatial coherence of the recorded signal.

These effects may locally elevate $C_{\textrm{eff}}$ even in the absence of a true burial transition. Consequently, the BI should be interpreted fundamentally as a proxy for cable–seafloor coupling irregularity, with burial representing a dominant but not exclusive control. At the study sites considered here, the close correspondence between elevated $C_{\textrm{eff}}$ intervals and independently mapped exposure or sedimentological boundaries suggests that burial-related coupling changes dominate the observed spatial structure, while rugosity-driven forcing variability likely contributes as a secondary effect.

While the present BI provides a robust and operationally useful indicator of coupling variability, an important next step is to extend such diagnostics beyond classification towards quantitative burial depth estimation. The current framework is designed to infer coupling state rather than burial depth directly; however, burial depth is expected to influence both attenuation and spatial regularity of the DAS response. This suggests that burial depth may be constrained using a broader class of functional analytic based regularity diagnostics, of which the Poincaré-style coherence ratio represents a particularly simple instance. For example, spatial total variation, curvature-based penalties, and multi-band spectral ratios may provide complementary constraints on coupling strength and sediment confinement. When combined with calibrated attenuation models and independent survey observations (e.g., ROV or diver measurements), these approaches may enable quantitative burial-depth estimation in future work.

Although the proposed method performs robustly across contrasting sites, several limitations and sensitivities should be recognised. The two-band spectral fingerprint used in this study was selected empirically through systematic evaluation of the DAS spectra at each site, guided by maximising spectral contrast and temporal stability. However, the optimal choice of frequency bands is not universal and may vary with environmental forcing, cable construction, sediment properties, coupling conditions, and DAS acquisition parameters such as gauge length and channel spacing. Despite this, the coherence-based framework itself is general and can be readily adapted to alternative frequency ranges. The method may also be influenced by instrumental artefacts, including slack sections, stick–slip behaviour, gain variations, or channel dropouts, which can introduce artificial coherence discontinuities resembling coupling transitions.

Alternative DAS processing approaches for subsea monitoring include classical array-based methods such as FK analysis, cross-correlation/coherence mapping, and beamforming^46,47^, as well as supervised learning-based classifiers and deep architectures such as CNN and Transformer models^49,50^ for event detection and segmentation. While these methods can provide rich inference capabilities, they may require more extensive tuning, training datasets, or higher computational overhead. The proposed Poincaré spectral coherence framework provides a complementary lightweight diagnostic based on spatial regularity constraints, requiring only short-time spectral estimation and local block statistics, see Table. 2

The spectral contrast exploited here is expected to depend on cable construction (armouring, stiffness, diameter), sediment type, and DAS acquisition parameters such as gauge length and channel spacing. Stiffer or heavily armoured cables may transmit higher-frequency vibration over longer distances, while soft sediment burial may produce stronger high-frequency attenuation. Similarly, longer gauge lengths may reduce sensitivity to short-wavelength vibration. These factors suggest that the optimal frequency bands and block length may require site-specific tuning, although the general Poincaré coherence formulation remains applicable.

The proposed approach differs from amplitude-only burial diagnostics in that it explicitly quantifies spatial regularity rather than absolute energy. Frequency-band-energy representations can reveal vessel signatures and attenuation trends, but they do not directly isolate coupling state under strongly time-varying forcing. By contrast, the Poincaré-style coherence formulation measures how consistently spectral fingerprints evolve across neighbouring channels, making it less sensitive to global changes in forcing amplitude and more sensitive to persistent coupling transitions. This complements recent work demonstrating coupling-dependent amplitude modulation and resonance shifts^38,39^ and offers a computationally lightweight alternative suitable for continuous real-time operation on long subsea arrays.

In practice, the workflow requires only short-time FFTs and block-wise algebraic statistics, allowing kilometre-scale burial maps to be produced in seconds on a single CPU core (Table 1), which is substantially less computationally demanding than deep-learning-based inference pipelines.

The computational simplicity of the proposed approach is an important practical advantage for operational deployment. As shown in Table. 1, once the spectral fingerprint is computed, all subsequent steps consist only of local block statistics and one-dimensional spatial operations with linear complexity. This enables burial-state maps to be generated continuously in near real time for kilometre-scale DAS arrays, without requiring computationally intensive inversion or learning-based training procedures. For example, as shown in Table 2, complete coupling-state maps for multi-kilometre cables can be generated in under 20 s on a single vCPU, enabling practical real-time deployment on large DAS datasets. This efficiency addresses a major challenge in DAS processing and distinguishes the proposed approach from many existing approaches including resonance-based coupling diagnostics, wave-type analysis, spectral energy mapping, and frequency-band-energy methods^10,38,39^

**Table 2:** Dataset characteristics and computational performance of the proposed $C_{\textrm{eff}}$ spectral-coherence method for two DAS datasets (EMEC Eday and OBSEA). All computations performed on Google Colab Pro (1 vCPU, 51 GB RAM).

Category	Metric	EMEC Eday (measured)	OBSEA (measured)
Geometry	Cable length (km)	2.207	5.815
	Channels	1083	4560
	Channel spacing (m)	2.040	1.275
Acquisition	Sampling rate (Hz)	1000	1000
	Duration (s)	540 (9 min)	200 (3.33 min)
	Samples per channel	540,000	200,000
$C_{\textrm{eff}}$ settings	Window length (s)	4	20
	Step size (s)	1	10
	Number of windows	539	19
	$C_{\textrm{eff}}$ segments	108	456
Performance	Total runtime (s)	16.78	16.68
	Time per window (s)	0.0311	0.878
	Time per km*·*hour (s)	50.7	51.6
	Time per channel*·*hour (s)	0.103	0.066
	CPU-hours per TB	1.99	1.27
Environment	Compute resource	Colab Pro (1 vCPU, 51 GB RAM)	Colab Pro (1 vCPU, 51 GB RAM)

The lightweight nature of the framework also enables future extensions, including adaptive band selection, multiscale coherence analysis, and integration with vessel detection or oceanographic monitoring pipelines. More broadly, coupling-aware DAS processing provides a valuable contextual layer for interpreting subsea observations. Because coupling variability modulates both amplitude and frequency response, the BI can be used to identify stable, well-coupled sections suitable for geophysical monitoring, as well as weakly coupled regions where signals are dominated by hydrodynamic or anthropogenic forcing. This supports improved interpretation of ocean noise fields and enhances the reliability of DAS-based ocean observing systems.

## Conclusion

This paper introduces a computationally lightweight and physically interpretable framework for real-time mapping of subsea cable burial state using DAS signals that provide differential phase or phase-rate measurements based on Rayleigh backscatter. By constructing band-integrated spectral fingerprints and exploiting a Poincaré-type smoothness inequality, we derived a normalised spectral coherence metric that quantifies spatial irregularity in the cable response. The resulting Burial Index (BI) enables identification of coupling transitions along the cable without requiring controlled sources, vessel transits, or prior geological calibration, and provides a robust indicator of buried, partially buried, exposed, and weakly supported cable segments.

Application to two independent field datasets (EMEC Eday and OBSEA) demonstrated that the proposed approach produces stable burial or coupling state profiles under highly non-stationary ocean forcing. At EMEC Eday, the BI revealed persistent spatial regimes that align with mapped lithological units and bathymetric context, supporting the interpretation that sedimentological transitions and seabed stiffness contrasts can modulate coupling behaviour and burial persistence. At OBSEA, the BI exhibited strong agreement with independently reported burial-state intervals from frogmen/diver and vessel observations, confirming that smoothness-based spectral coherence provides an effective burial proxy in realistic shallow-water environments.

Beyond burial inference, the results emphasise that inequality-based coherence metrics provide a general mathematical tool for quantifying how mechanical boundary conditions shape the recorded DAS field. Persistent BI anomalies may reflect not only burial transitions but also broader cable–seafloor interaction effects, including free-span vibration, intermittent sediment contact, seabed rugosity, bathymetric roughness, and spatially heterogeneous hydrodynamic forcing. This highlights the potential for DAS to support a more general cable–seafloor coupling classification capability, in which burial is treated as one component of a broader, quantifiable environmental state.

Future work should focus on extending the present framework beyond qualitative burial-state mapping toward quantitative estimation of burial depth and coupling strength. The computational efficiency of functional-inequality-based regularity measures provides a particularly promising pathway: spatial total variation, curvature (bending-energy) constraints, and related smoothness inequalities can be evaluated rapidly and offer physically interpretable diagnostics of confinement strength. When combined with multi-band attenuation models and calibrated against independent survey observations (e.g., ROV or diver burial measurements), such regularity diagnostics could enable burial-depth inference rather than purely categorical classification. Resonance-based proxies and wave-type dependent coherence attributes may further complement this approach by providing independent sensitivity to coupling stiffness and sediment confinement. Additional priorities include explicitly incorporating bathymetry and rugosity into interpretation to separate burial-driven coupling changes from forcing heterogeneity, and integrating the workflow into operational DAS processing pipelines for scalable real-time monitoring of subsea infrastructure and improve the reliability of environmental inference in DAS-enabled ocean observing systems.

## Figures and Tables

**Fig. 15:**
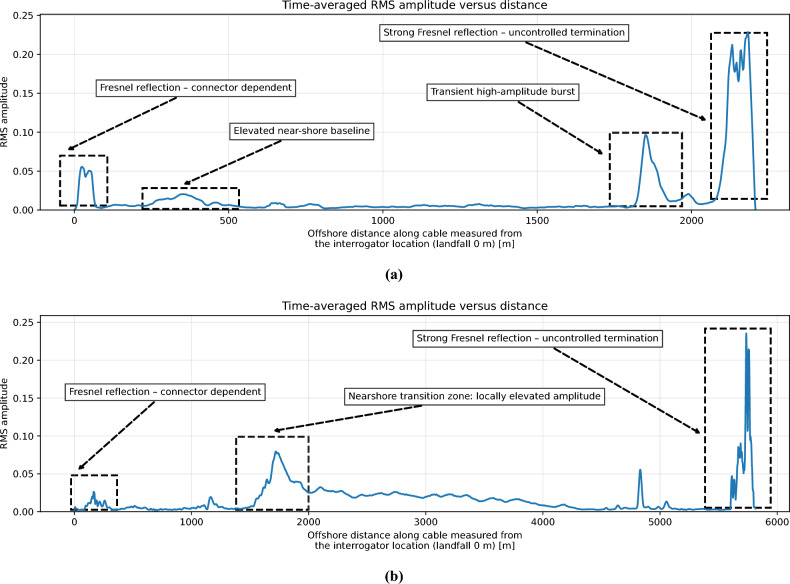
Time-averaged RMS amplitude as a function of distance along the cable for two DAS datasets. (a) EMEC Eday Cable 4 (Orkney, UK), showing an elevated near-shore baseline associated with shallow-water forcing, followed by reduced offshore amplitude and intermittent transient high-amplitude bursts occurring upstream of the Fresnel reflection region. The offshore end exhibits strong Fresnel reflections due to the uncontrolled termination. (b) OBSEA coastal observatory (Barcelona, Spain), showing a distinct nearshore transition zone characterised by locally elevated amplitude, consistent with variable coupling conditions, followed by relatively stable offshore behaviour and Fresnel reflection effects at the cable termination.

**Fig. 16:**
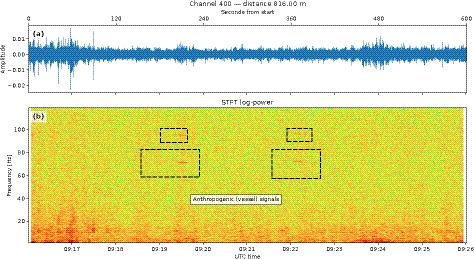
Spectrogram of single channel belong to the EMEC Eday dataset.

**Algorithm 1:**
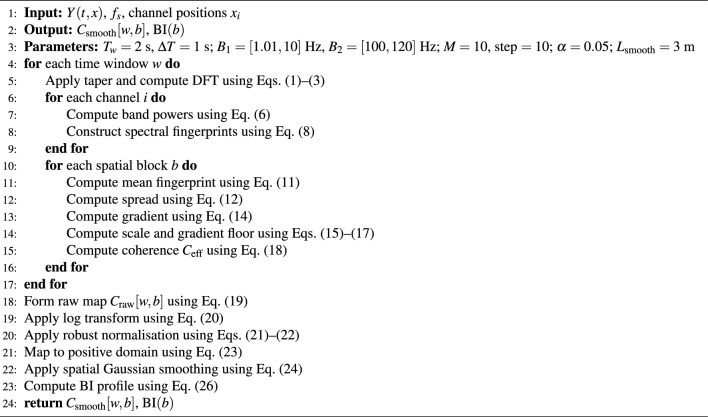
Computation of BI from smoothed normalised $C_{\textrm{eff}}$ .

## Data Availability

The datasets generated and analysed during this study are available from the corresponding author on request. Due to its large size, the dataset is not available on a public repository.

## A Appendix: Theoretical definition of frequency band energy (FBE)

FBE provides a measure of the signal power contained within a specified frequency interval. For a windowed and tapered discrete-time signal segment $y_i^{(w)}[n]$, $n = 0, \dots , N_t^{(w)}-1$, the tapered signal is defined as

27 $$\begin{aligned} \tilde{y}_i^{(w)}[n] = y_i^{(w)}[n]\, h[n], \end{aligned}$$

where *h*[*n*] denotes the Hanning window introduced in Eq. (1).

The discrete Fourier transform (DFT) of the tapered signal is then given by

28 $$\begin{aligned} \hat{y}_i^{(w)}[k] = \sum _{n=0}^{N_t^{(w)} - 1} \tilde{y}_i^{(w)}[n]\, e^{-j 2\pi nk/N_t^{(w)}}, \qquad k = 0, \dots , N_t^{(w)}-1. \end{aligned}$$

Let $S_{yy}(f)$ denote the power spectral density (PSD) of the signal. The continuous definition of FBE over a frequency band $B = [f_1, f_2]$ is

29 $$\begin{aligned} \textrm{FBE}(B) = \int _{f_1}^{f_2} S_{yy}(f)\, df. \end{aligned}$$

In practice, the PSD is approximated using a discrete spectral estimate derived from the DFT. For a sampling frequency $f_s$, the discrete frequency grid is

30 $$\begin{aligned} f_k = \frac{k}{N_t^{(w)}} f_s, \end{aligned}$$

and the band-limited energy can be written as

31 $$\begin{aligned} \textrm{FBE}(B) \approx \sum _{k \in K_B} P_i^{(w)}[k]\, \Delta f, \end{aligned}$$

where $K_B$ is the set of indices such that $f_k \in [f_1, f_2]$, $P_i^{(w)}[k]$ is a spectral power estimate, and $\Delta f = \frac{f_s}{N_t^{(w)}}$.

Using the periodogram approximation,

32 $$\begin{aligned} P_i^{(w)}[k] = \frac{1}{N_t^{(w)} f_s} \left| \hat{y}_i^{(w)}[k] \right| ^2, \end{aligned}$$

the band energy becomes

33 $$\begin{aligned} \textrm{FBE}(B) \approx \frac{1}{N_t^{(w)} f_s} \sum _{k \in K_B} \left| \hat{y}_i^{(w)}[k] \right| ^2 \, \Delta f. \end{aligned}$$

Neglecting constant scaling factors (which do not affect relative comparisons across space and time), a commonly used discrete approximation is

34 $$\begin{aligned} \textrm{FBE}(B) \propto \sum _{k \in K_B} \left| \hat{y}_i^{(w)}[k] \right| ^2. \end{aligned}$$

This formulation is consistent with Parseval’s theorem, which establishes the equivalence between the total energy of a signal in the time domain and the sum of squared magnitudes of its Fourier coefficients in the frequency domain. Restricting this summation to a subset of frequency bins therefore yields a physically meaningful measure of band-limited signal energy.

## B Appendix: Relationship between MAD and standard deviation

For a Gaussian random variable $X \sim \mathscr {N}(\mu , \sigma ^2)$, the Median Absolute Deviation (MAD) is defined as

$$\textrm{MAD} = \textrm{median}(|X - \textrm{median}(X)|).$$

For the normal distribution, the MAD is related to the standard deviation by

$$\textrm{MAD} = \sigma \cdot \Phi ^{-1}(0.75),$$

Here, $\Phi ^{-1}(.)$ denotes the inverse cumulative distribution function (quantile function) of the standard normal distribution, which maps cumulative probabilities to their corresponding standard-normal variates^42^. Since

$$\Phi ^{-1}(0.75) \approx 0.67449,$$

the standard deviation can be approximated as

$$\sigma \approx \frac{\textrm{MAD}}{0.67449} \approx 1.4826 \cdot \textrm{MAD}.$$

Thus, the factor 1.4826 is a consistency constant that allows the MAD to be expressed on the same scale as the standard deviation under Gaussian assumptions.

## C Appendix: Supplementary site overviews for DAS datasets

The time-averaged RMS amplitude profiles shown in Fig. 15 provide a complementary, distance-resolved summary of the raw DAS phase-rate response for both the EMEC and OBSEA datasets (see Figs. 3 and 9). These profiles capture persistent spatial trends in signal amplitude that are consistent with the broadband behaviour observed in the raw time–distance heatmaps. In particular, elevated nearshore amplitudes and localised transitions correspond to regions of stronger environmental forcing and variable cable–seafloor coupling, while reduced or stable offshore amplitudes reflect more uniform conditions. The dashed and solid annotated regions denote connector-dependent and strong Fresnel reflections, respectively, with the offshore termination producing the dominant response in both datasets, whereas the near-interrogator contribution is comparatively weak in the OBSEA case. Overall, the RMS representation provides a compact and intuitive diagnostic that supports and validates the interpretation of spatial coupling variability presented in the main analysis.

## D Appendix: Time frequency representation of single channel over the time

To further clarify the selection of frequency bands, Fig. 16 includes a representative single-channel time–frequency spectrum from EMEC Eday dataset. In addition to the general separation between low- and high-frequency regimes, the figure highlights distinct narrow-band features in the 60–100 Hz range. Specifically, between approximately 09:19–09:20 and 09:22–09:23 UTC, clear narrow-band spectral lines are observed. These features correspond to a vessel crossing event recorded at this channel, manifesting as temporally localised, frequency-constrained energy. Such narrow-band signatures are characteristic of anthropogenic sources (e.g., vessel machinery and propeller harmonics) and illustrate the type of high-frequency, intermittent excitation captured within the selected upper frequency band.

## E Appendix: Algorithmic implementation of the proposed framework

This appendix provides a concise algorithmic summary of the proposed Poincaré spectral coherence framework used to compute the smoothed coherence map and the BI. The algorithm directly corresponds to the mathematical formulation presented in Section Methodology, with each step referencing the relevant equations for clarity and reproducibility.

The procedure operates on DAS measurements *Y*(*t*, *x*) and consists of four main stages: (i) spectral fingerprint construction using band-limited energy, (ii) block-wise computation of the Poincaré-style spatial coherence $C_{\textrm{eff}}$, (iii) robust log–MAD normalisation, and (iv) spatial Gaussian smoothing followed by temporal aggregation to obtain the BI profile.

All parameter values used in this study are explicitly specified within the algorithm to ensure full reproducibility.
